# Current status of *Stenothoe gallensis* (Amphipoda, Stenothoidae): Towards the re-appraisal of the species complex with new records from Greece and challenges arising from intraspecific morphological variation

**DOI:** 10.3897/BDJ.14.e199461

**Published:** 2026-07-07

**Authors:** Maria Lampa, Nafsika Papageorgiou, Dimitra Chatzivasileiou, Panagiotis D. Dimitriou, Stefania M. Manolaki, Ioannis Karakassis, Giorgos Chatzigeorgiou, Wanda Plaiti

**Affiliations:** 1 Department of Biology, University of Crete, Heraklion Crete 70013, Greece Department of Biology, University of Crete Heraklion Crete 70013 Greece; 2 Institute of Oceanography, Hellenic Centre for Marine Research, Gournes Crete 71003, Greece Institute of Oceanography, Hellenic Centre for Marine Research Gournes Crete 71003 Greece; 3 Department of Agricultural Development, Agri-Food & Natural Resources Management, National and Kapodistrian University of Athens, Evripos Complex, GR 34400, Greece Department of Agricultural Development, Agri-Food & Natural Resources Management, National and Kapodistrian University of Athens Evripos Complex, GR 34400 Greece; 4 Institute of Marine Biology, Biotechnology and Aquaculture (IMBBC), Hellenic Centre for Marine Research, Gournes Crete, 71003, Greece Institute of Marine Biology, Biotechnology and Aquaculture (IMBBC), Hellenic Centre for Marine Research Gournes Crete, 71003 Greece

**Keywords:** biodiversity, morphometry, NIS, non-indigenous species, taxonomy

## Abstract

**Background:**

*Stenothoe gallensis* Walker, 1904 is one of the most widely reported species, with records from various localities across the globe, including the Indian, the Pacific and the Atlantic Oceans. Nevertheless its cosmopolitan distribution has increasingly been questioned and, in recent years, the taxon has been established as a species complex whose members are recognised for their invasion potential.

**New information:**

This study revealed the presence of specimens belonging to the *S. gallensis* complex, which closely matched the original description of *Stenothoe irakiensis* Salman, 1985. This species is currently considered a junior synonym of *S. gallensis*. A thorough examination of key linear and meristic morphological characters across sexes and maturity stages revealed significant intraspecific variation in several characters commonly used for species diagnosis. This size-, sex- and maturity-dependent variation within the population, when combined with incomplete or conflicting species descriptions from different localities, introduces uncertainty regarding the reliability of the diagnostic features that were used to identify members of the *Stenothoe gallensis* complex. This, in turn, complicates the interpretation of previous records and the delimitation of taxa within the complex. These findings provide a compelling rationale for a comprehensive re-assessment of records attributed to the *S. gallensis* complex, underscoring the necessity for an integrative taxonomic revision incorportating both morphological and molecular data from material collected at the designated type localities.

## Introduction

The genus *Stenothoe* Dana, 1852, is one of the 48 currently accepted genera within the family Stenothoidae and comprises 63 accepted species amongst its 93 direct children, seven of which are considered nomina dubia (WoRMS 2026). It is a widely distributed genus, with 8,476 occurrence records worldwide according to GBIF (2026), many of which have been documented in the Mediterranean Sea, where 14 species have been reported (Martínez-Laiz et al. 2020). This genus has been found in a variety of habitats and communities, including fouling species such as *Stenothoe valida* Dana, 1853 and the recent invader *Stenothoe georgiana* Bynum & Fox, 1977. *S. georgiana* has been found in ports (Ulman et al. 2017, Ferrario et al. 2018) and aquaculture facilities (Fernandez-Gonzalez and Sanchez-Jerez 2017) and generally in association with artificial substrates such as ropes, pontoons, wheels, buoys and vessel hulls (Martínez-Laiz et al. 2020).

Amongst the species of *Stenothoe*, one of the most widely recorded is *Stenothoe gallensis* Walker, 1904, which has been reported from numerous localities worldwide and is considered to have a cosmopolitan distribution. At present, a total of 567 records of the species are currently available on the GBIF dataset (GBIF 2026). The cosmopolitan nature of the species has been questioned and, in recent years, it has been established as a species complex, which has yet to be conclusively resolved (Zettler 2021, Azman 2023). All species belonging to *Stenothoe gallensis* species complex are recognised for their invasion potential (Martínez-Laiz et al. 2020). Even until recently, the species *Stenothoe gallensis* Walker, 1904 has been considered an alien species in the Mediterranean Sea (Zenetos et al. 2018), but its presence has been questioned, since the Mediterranean material examined by Krapp-Schickel (2013) was found to actually belong to *Stenothoe cattai* Stebbing, 1906, another member of the species complex.

Later, Krapp-Schickel (2015) revised some of the species in the *Stenothoe gallensis* species complex, added four new species and provided a worldwide identification key for the members of the genus *Stenothoe*. Following the revision by Krapp-Schickel (2015) and subsequent descriptions, eleven species are currently included in the *Stenothoe gallensis* species complex: *Stenothoe andamanensis* Krapp-Schickel, 2015, *Stenothoe cattai* Stebbing, 1906, *Stenothoe clavetta* Krapp-Schickel, 2015, *Stenothoe crenulata* Chevreux, 1908, *Stenothoe dentirama* Hirayama & Takeuchi 1993, *Stenothoe gallensis* Walker, 1904, *Stenothoe himyara* Krapp-Schickel, 2015, *Stenothoe irinae* Marin & Sinelnikov, 2018, *Stenothoe irakiensis* Salman, 1985 (considered as a junior synonym of *S. gallensis*), *Stenothoe senegalensis* Krapp-Schickel, 2015 and *Stenothoe uncinifera* Mateus and Mateus 1966 (which is regarded as nomen dubium).

*Stenothoe irakiensis* was described by Salman in 1985, who stated that it differs from *Stenothoe gallensis* Walker, 1904 by the presence of a sharp ventral spur on the peduncle of uropod 1. In the re-description of *S. gallensis*, carried out by Krapp-Schickel (2015), the author reported the same character in the material examined and consequently treated *S. irakiensis* as a junior synonym of *S. gallensis*. The type locality of *S. irakiensis* is the Arabian Gulf, while the one of *S. gallensis* is Sri Lanka (Walker 1904). However, the latter has been frequently recorded in the Mediterranean Sea, including the Greek waters. *S. irakiensis* is known only from its type locality, the Arabian Gulf and it had not been previously reported from the Mediterranean Sea, even before its proposed synonymisation with *S. gallensis* in 2015.

In the context of this study, several individuals morphologically corresponding to the description of *S. irakiensis* (Salman 1985) were collected from aquaculture farms in Greece. The main objective of this study was to investigate which of the morphological characters used in comparisons amongst members of the *S. gallensis* species complex, either individually or in combination, are most reliable for species discrimination, while accounting for intraspecific variation not previously investigated. Additionally, the study aims to re-assess the taxonomic status of *S. gallensis* complex through examination and description of newly-collected records from Greece, as well as to identify and clarify inconsistencies in the existing literature. Based on these results, a provisional morphological taxonomic framework is proposed for interpreting variability within the *S. gallensis* complex, to facilitate future integrative taxonomic research and improve understanding of this species complex.

## Materials and methods

### Study area and sample collection

**Figure 1.**
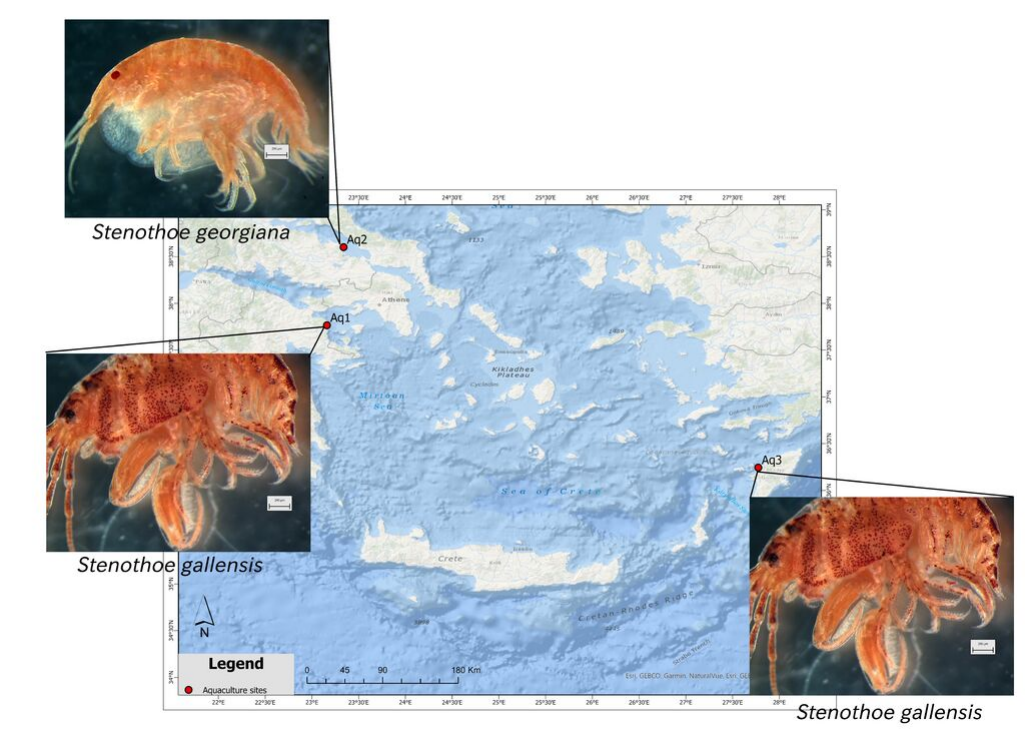
Map showing the locations of the three aquaculture sampling sites and the associated *Stenothoe* species collected from each site.

The present study was conducted in the context of detecting non-indigenous species, with a specific focus on specimens of the genus *Stenothoe*, particularly the *Stenothoe gallensis* complex, recovered from samples collected in July and November 2021 from three aquaculture farms in Greece. Samples containing *Stenothoe* individuals were collected from aquaculture sites Aq1 (37°45'55.35"N, 23°9'39.69"E) located near Korfos in Saronikos Gulf; Aq2 (38°36'3.83"N, 23°20'19.92"E), located in northern Evoikos Gulf; and Aq3 (36°14'38.9"N, 27°46'28.9"E) located in the island of Rhodes. The samples investigated in the present study correspond to aquaculture sites Aq1, Aq2 and Aq3 as reported in Chatzivasileiou et al. (2022). A map showing the locations of these sites is also provided (Fig. 1). Quantitative samples were collected by scraping organisms from mussel and oyster spat-collecting ropes with a metal blade along their lengths (10 cm sampling surface) at a depth range of 1-6 cm. One sample from each depth was hand-collected and presereved in 4% formaldehyde. Samples were also collected from all three aquaculture sites, but only specimens from Aq1 and Aq3 belong to the *S. gallensis* species complex. *Stenothoe* specimens from Aq2 (northern Evoikos Gulf) were identified as *S. georgiana*, which is a recent invader that has established a widespread distribution in many regions (Martínez-Laiz et al. 2020) *S. gallensis* individuals were identified, based on a combination of existing literature (Walker 1904, Krapp-Schickel 1976, Ruffo 1993, Lecroy 2011, Krapp-Schickel 2013, Krapp-Schickel 2015, Marin and Sinelnikov 2018, Azman 2023). *S. georgiana* was also identified, based on recent literature (Bynum and Fox 1977, Pérez-Schultheiss and Ibarra 2017, Martínez-Laiz et al. 2020).

### Morphometrical and Statistical analyses

A total number of 81 specimens assignable to the *S. gallensis* species complex, including 50 male and 31 female specimens from Aq1 and Aq3, were measured for the collection of morphometric data. Male specimens included both younger individuals (29 specimens) and hyperadults (21 specimens). The distinction of hyperadults from immature individuals and subadults was based on: (a) the comparatively larger body size and stronger gnathopod 2 - Gn2 (refer to description for hyperadult males and for subadult/young males below) - with hyperadults possessing a longer and more robust dactylus; (b) the presence of a row of distinct setae on the inner margin of Gn2 dactylus and regular crenulations of Gn2 merus of hyperadults (see also description below).

**Figure 2.**
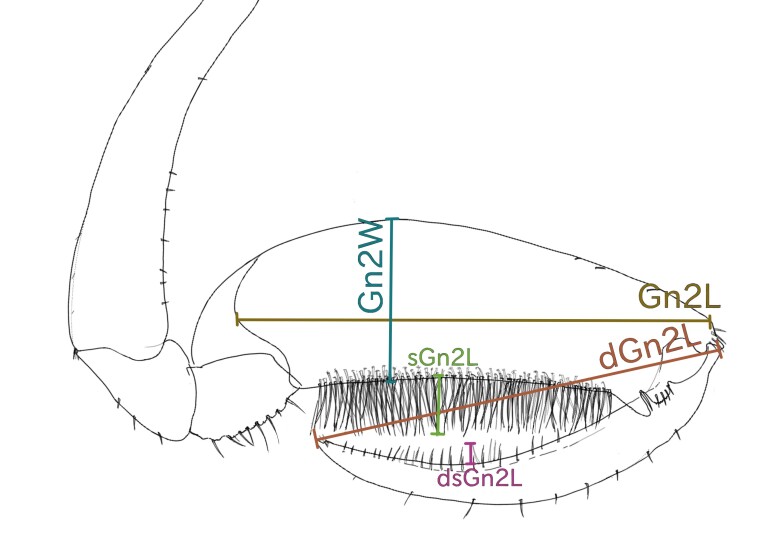
Linear measurements of hyperadult male gnathopod 2 (Gn2) of *Stenothoe gallensis* (Gn2L - Gn2 propodus length; Gn2W- Gn2 propodus width; dGn2L - Gn2 dactylus length; sGn2L- length of setae on the palmar margin of Gn2; dsGn2L - length of setae on the inner margin of Gn2 dactylus).

The measurements were taken under an Olympus SZX9 stereoscope and with the use of an Olympus EP50 camera. To measure and compare specimen size, total length (TL) was measured as the sum of curvilinear dorsal body length (BL) and curvilinear length of antennae (AL). In addition, linear variables measured were: body width (BW); gnathopod 2 propodus length (Gn2L); gnathopod 2 propodus width (Gn2W); gnathopod 2 dactylus length (dGn2L); length of setae on the of palmar margin of gnathopod 2 (sGn2L); and length of setae on inner margin of gnathopod 2 dactylus (dsGn2L) (Fig. 2).

Key meristic identification characters examined were: (1) the number of robust setae on the proximal telson margin; (2) the number of marginal unpaired robust setae on the peduncle of male uropod 3 - U3; (3) the number of distal robust setae on the U3 peduncle; and (4) the number of distal robust setae on U3 article 1. Additionally, the U3 male ratio to peduncle to article 1 to article 2 was measured. These characters were selected for the analysis as they are key identification characters used for the separation of the species. The selected characters are presented in comparative tables across species of the complex in recent literature (Krapp-Schickel and Sket 2015, Marin and Sinelnikov 2018, Azman 2023). For example, Krapp-Schickel (2015) identifies the number of pairs of robust setae on the telson as a diagnostic character and characterises the setae on the palm of Gn2 as short or long and as few or absent, whereas Marin and Sinelnikov (2018) compare the armature of uropod 3 (U3), as well as the length of dactylus relative to the length of posterior palmar margin of Gn2. Additional morphological characters regarded as diagnostic for species discrimination are presented in Suppl. material 2 - Table S1.

Spearman rank (Spearman 1904) correlations were calculated amongst morphometric variables. Subsequently, scatter plots were created to visualise the relationships between the male Gn2 propodus length, male Gn2 dactylus length and body size. Differences between male hyperadults and younger male individuals (subadults and immature males) were tested using Mann-Whitney U tests (Elliot 1977). Finally, a Principal Components Analysis was performed on the normalised (z-standardisation) linear and meristic identification characters (PCA, Clarke and Gorley (2015)) to explore patterns of morphological variation and to identify traits contributing most to differences amongst male hyperadults, younger male individuals and females. All statistical analyses were performed in R (version 4.5.1 - R Development Core Team (2025)) and PRIMER-E v.7 (Clarke and Gorley (2015)).

The dataset of morphometric variables and their measured values is available in the Supplementary Material (Suppl. material 3 - Tables S3 and S4).

### Records of Stenothoe gallensis

Mediterranean records of *S. gallensis* were compiled from the literature, including research articles indexed in the Scopus database and from GBIF (GBIF 2026). Worldwide records from 2015 onwards, following the re-description of *S. gallensis* and revision of the species complex by Krapp-Schickel (2015), were also compiled from both literature and GBIF. All data were subsequently visualised using ArcGIS Pro 3.0.3. The compiled dataset, along with the corresponding references, is provided as supplementary material (Suppl. material 4).

## Taxon treatments

### Stenothoe gallensis

Walker, 1904

90C8DF67-F950-5567-8216-07C49B98B536

*Stenothoe gallensis* Walker, 1904: 261–62, pl. 3, fig. 19 (Sri Lanka), Nayar, 1967: 144–45, fig. 5e (Sri Lanka), Krapp-Schickel 1976: 15-18, figs. 14-16, Krapp-Schickel 2015: 8-10 (Dar es Salaam, Tanzania).
*Stenothoe irakiensis* Salman, 1985: 244–250, figs. 1–4 (Arabian Gulf).

#### Materials

- **Type status:** Other material. **Occurrence:** individualCount: 44; sex: male; occurrenceID: 08B4A85C-A828-5713-9DFA-19AB808EAAD3; **Taxon:** taxonID: urn:lsid:marinespecies.org:taxname:103163; scientificName: Stenothoe gallensis; **Location:** country: Greece; locality: Korfos; verbatimLocality: Aq1; verbatimLatitude: 37.765375; verbatimLongitude: 23.161024; **Identification:** identifiedBy: Maria Lampa; dateIdentified: 2025; **Event:** eventDate: 2021
- **Type status:** Other material. **Occurrence:** individualCount: 23; sex: female; occurrenceID: 100596F1-3FA0-5E81-807D-C7B58F95E68D; **Taxon:** taxonID: urn:lsid:marinespecies.org:taxname:103163; scientificName: Stenothoe gallensis; **Location:** country: Greece; locality: Korfos; verbatimLocality: Aq1; verbatimLatitude: 37.765375; verbatimLongitude: 23.161024; **Identification:** identifiedBy: Maria Lampa; dateIdentified: 2025; **Event:** eventDate: 2021
- **Type status:** Other material. **Occurrence:** individualCount: 6; sex: male; occurrenceID: 73481607-D4F3-56CB-B907-D26177F1D633; **Taxon:** taxonID: urn:lsid:marinespecies.org:taxname:103163; scientificName: Stenothoe gallensis; **Location:** country: Greece; locality: Rhodes; verbatimLocality: Aq3; verbatimLatitude: 36.244136; verbatimLongitude: 27.774688; **Identification:** identifiedBy: Maria Lampa; dateIdentified: 2025; **Event:** eventDate: 2021
- **Type status:** Other material. **Occurrence:** individualCount: 7; sex: female; occurrenceID: 238F4351-C521-59E9-8949-B355037BBA7A; **Taxon:** taxonID: urn:lsid:marinespecies.org:taxname:103163; scientificName: Stenothoe gallensis; **Location:** country: Greece; locality: Rhodes; verbatimLocality: Aq3; verbatimLatitude: 36.244136; verbatimLongitude: 27.774688; **Identification:** identifiedBy: Maria Lampa; dateIdentified: 2025; **Event:** eventDate: 2021

#### Description

##### Hyperadult males

**Figure 3.**
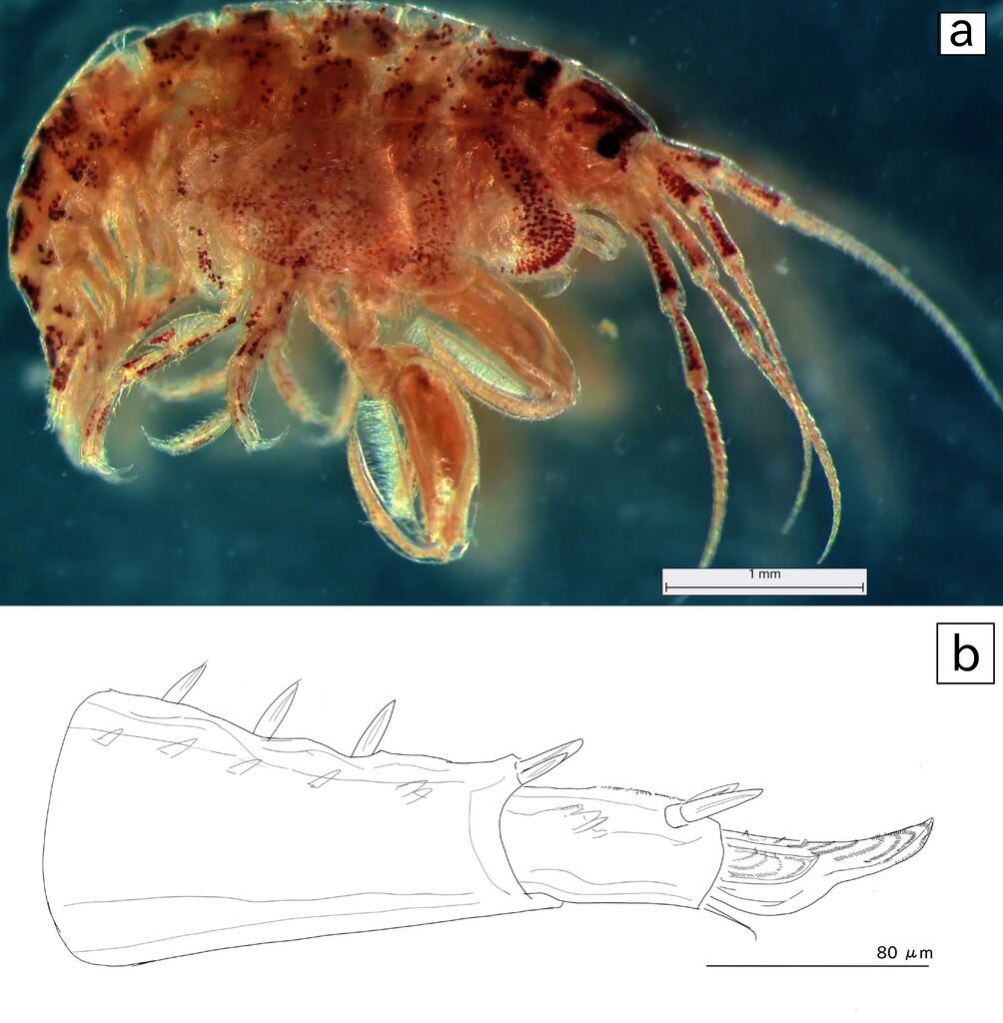
*Stenothoe gallensis* hyperadult male: **(a)** photograph of complete specimen; total curvilinear length: 6.73 mm; **(b)** linear drawing of detail of uropod 3.

**Figure 4.**
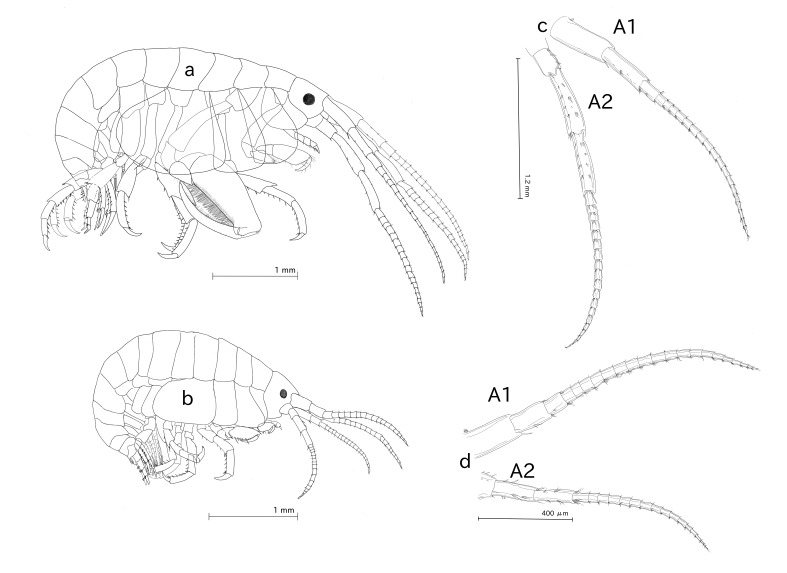
*Stenothoe gallensis*. Line drawings of a hyperadult male **(a)** and female **(b)** (total curvilinear length: male, 6.65 mm; female, 5.0 mm); **(c)** antennae 1 and 2 (A1, A2) of the hyperadult male; **(d)** antennae 1 and 2 (A1, A2) of the female.

Total mean curvilinear length (TL) of hyperadult 7.33 ± 1.18 (Figs 3, 4), with the largest examined individual reaching 11 mm. Total mean body width (BW) 1.34 ± 0.22. Pigmentation pattern on well-preserved specimens with punctate spots scattered across the cuticle and appendages, densely concentrated dorsally and more sparsely scattered distally.

Body: Mean BL 5.01 ± 0.97 mm. Coxa 2 and 3 posterior margins straight; anterior margins rounded. Coxa 4 entire, without a notch on the anterodistal corner.

Eyes: Darkly pigmented, relatively small and rounded, with a mean diameter of 101 ± 18 μm and a mean perimeter of 353 ± 59 μm.

Antennae (Fig. 4a and c): Subequal in length, mean curvilinear length (AL) 2.32 ± 0.32 mm; approximate length 0.5x body length. A1 ratio of peduncular articles 1-3 is 1.2:1:0.4, flagellum typically with 22-24 articles; A2 ratio of peduncular articles 4-5 is 1:0.8, with article 3 being 0.4x article 4; flagellum typically with 18-22 articles: this number appears to be size dependent, with smaller individuals showing fewer articles, with the lowest number of flagellum articles observed being 11 for A1 and nine for A2. Accessory flagellum not observed. A1 flagellum with a distal aesthetasc on each segment. A1 peduncular article 1 typically bearing one distal robust seta on the posterior margin. A2 peduncular article 3 typically with 3–4 robust setae on the anterior margin and one distal robust seta on the posterior margin; peduncular article 4 typically bearing 1-2 robust setae on the anterior margin.

**Figure 5.**
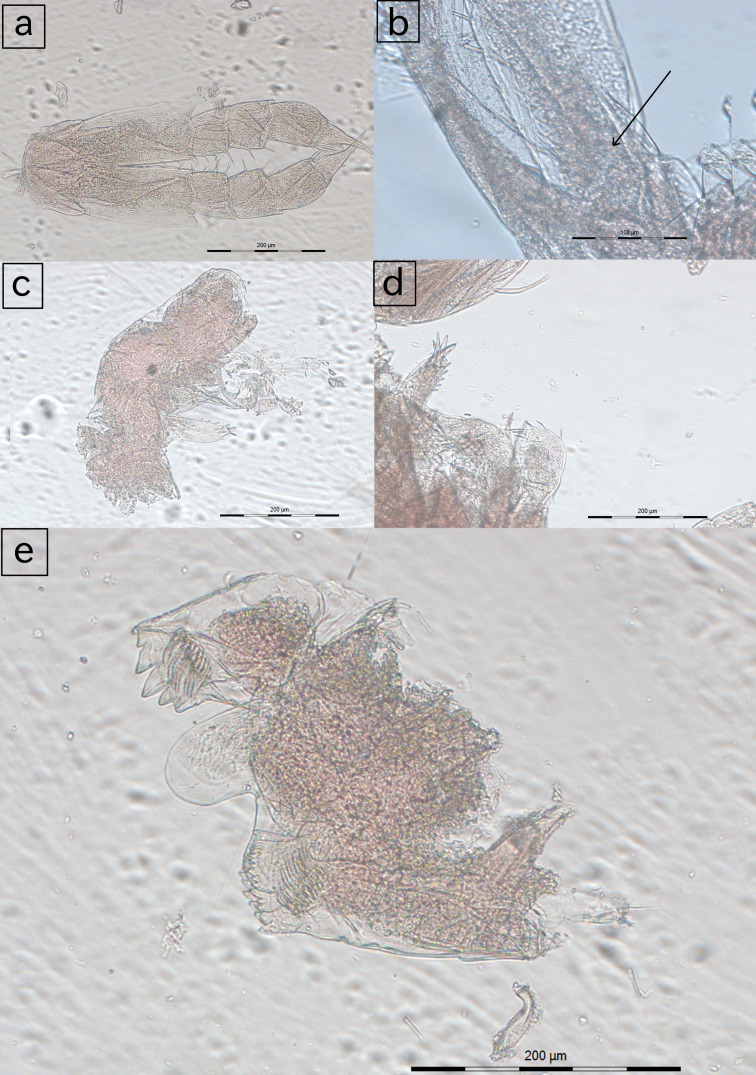
Mouthparts of *Stenothoe gallensis*. **a** maxilliped; **b** inner plate of maxilliped (pointed by black arrow); **c** palp of maxilla I; **d** maxilla I and maxilla II; **e** upper lip and right and left mandibles.

**Figure 6.**
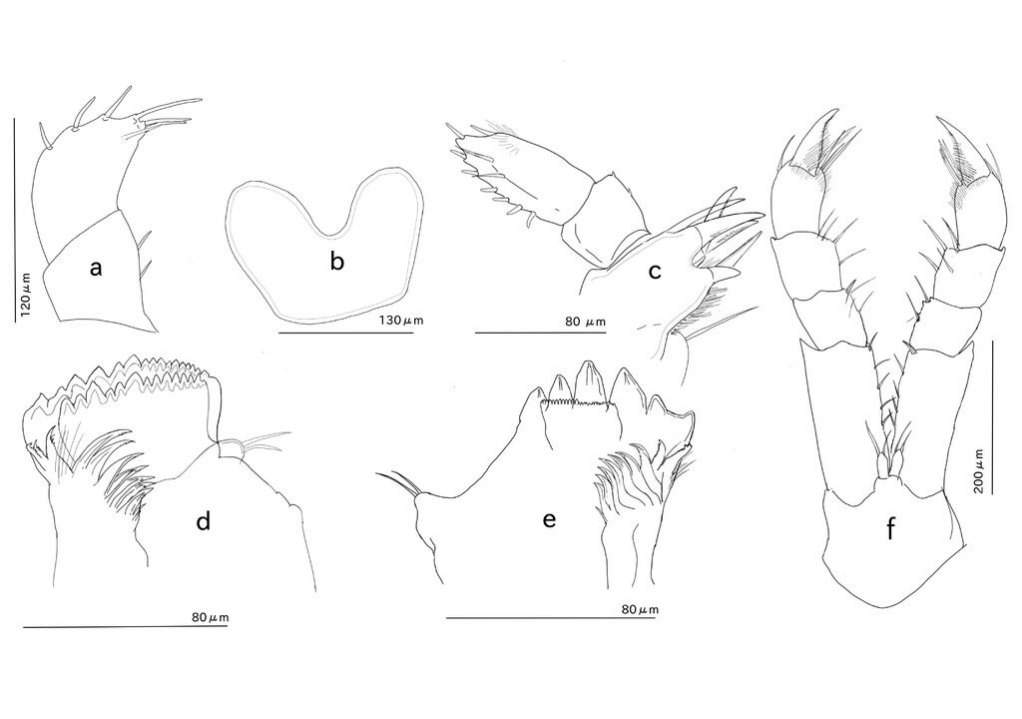
Mouthparts of *Stenothoe gallensis*: **a** maxilla II; **b** upper lip; **c** maxilla I; **d** left mandible; **e** right mandible; **f** maxilliped.

Mouthparts: Right mandible with strong incisor process and lacinia mobilis relatively narrow with finely serrated distal part; incisor process typically with five teeth; distal tooth typically with two distal cusps; lacinia mobilis ca. 16-20 teeth; with two thicker dentate setae nearest incisor and 7-9 simpler dentate setae further away (Fig. 5e and Fig. 6e). Left mandible incisor process and lacinia mobilis broad, minutely denticulate with relatively sharp teeth; incisor process ca. 16-18 teeth and lacinia mobilis ca. 14-16 teeth; with three thicker dentate setae nearest incisor and 7-8 further away (Fig. 5e and Fig. 6d). Mandibles without palp, but with a small protrusion with two setae. Maxilliped (Fig. 5a and Fig. 6f) palp 4-articulate; article 4 (dactylus) a little longer (1.25x) than article 3; article 3 with one stiff seta at distal end of inner margin and long simple setae distally on both inner and outer margins; ischium (basal article of endopod) longer (1.4x) than palp articles 1 and 2 combined, with a pointed distolateral edge; short setae on inner margins of palp articles 1, 2 and ischium, no setae on outer margins; inner plate of maxillipeds (Fig. 5b) reduced with two apical setae; length of inner plate 0.2x length of maxilliped palp ischium; outer plate lacking. Upper lip (Fig. 5e and Fig. 6b) lobes subequal. Maxilla I (Fig. 5c, d and Fig. 6c) inner plate with one long seta; outer plate with six robust setae - two strong and cuspidate, three serrated with multiple, thin serrations, one slightly less robust, inner margin with a patch of thin simple setae; palp 2-articulate, distal article with a row of 5-8 setae on the outer margin and typically with 2-4 setae positioned slightly inwardly; distal article subequal in width to proximal article, but narrowing towards the apex. Plates of maxilla II (Fig. 5d and Fig. 6a) separated; outer plate (distal article) with six setae on outer margin and typically one seta positioned slightly inwardly; inner plate (proximal article) typically with 2-4 setae; outer plate subequal in width, but 1.2 - 1.5x length of inner plate.

**Figure 7.**
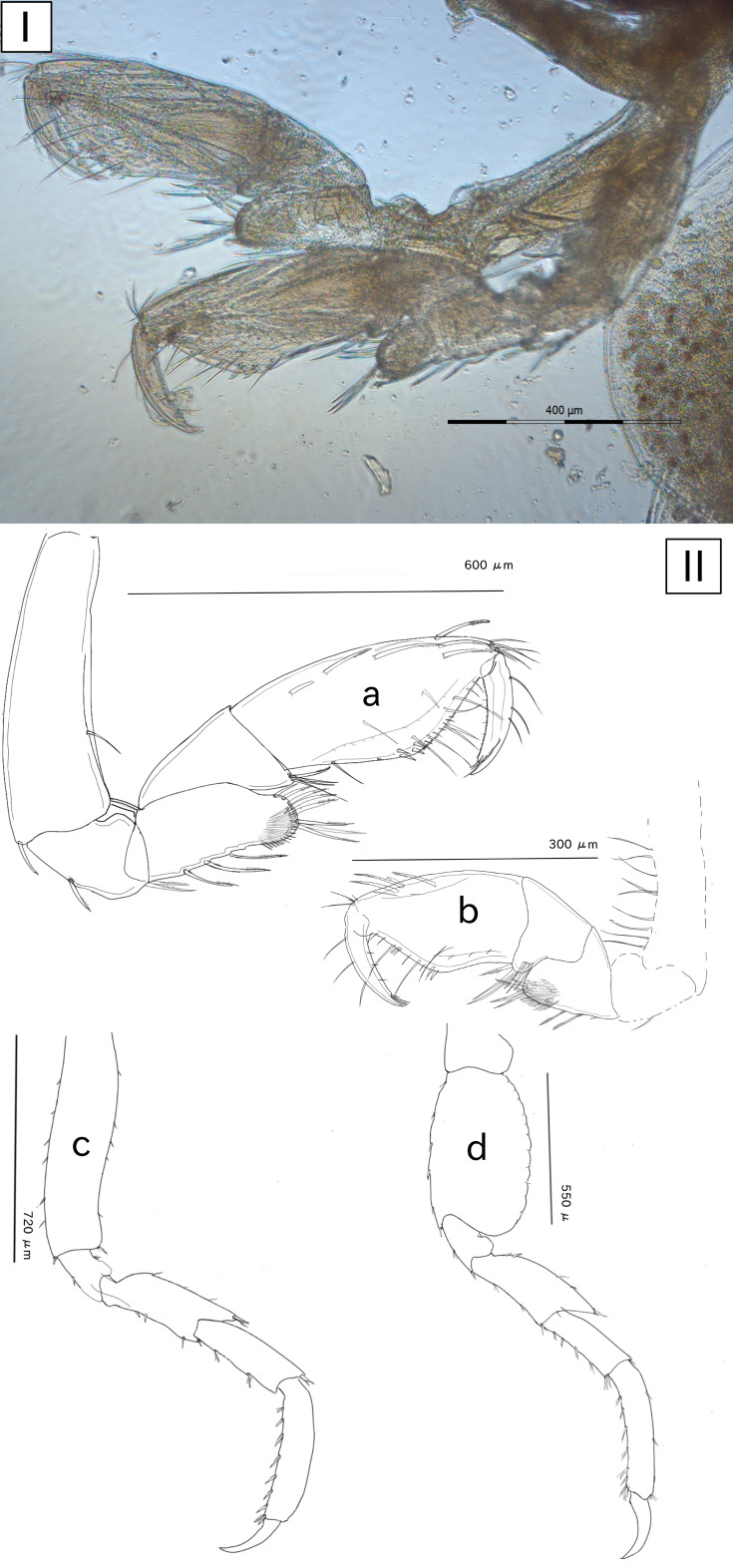
Appendages of *Stenothoe gallensis* (gnathopod 1, peraeopods 3 and 7): (I) photograph of gnathopods of the first pereopod (Gn1) of hyperadult male; (II) Line drawings of *Stenothoe gallensis*: **a** male gnathopod 1; **b** female gnathopod 1; **c** male peraeopod 3; **d** male peraeopod 7.

Gnathopod 1 - Gn1: Gn1 (Fig. 7I and IIa) propodus elongate, longer than carpus and merus, length of propodus approximately longer than 2x width; palmar margin subequal in length to posterior margin, beset with 4-6 robust setae, 5-6 simple long setae and a row of short setae; anterior margin with 3-6 stiff, comb-like setae along the length of the margin and a patch of 5-7 simple setae, located antero-distally; dactylus outer margin with three long simple setae, inner margin with minute setae and three long simple setae inserted near the tip of the dactylus; carpus triangular, posterodistal corner with a crown of 7-8 long comb-like and 2-3 simple setae; merus subrectangular, elongate, free distal lobe with a patch of simple, fine setae and a crown of 8-10 comb-like setae; 3-5 comb-like setae along the length of posterior margin of merus; ischium with posterior margin bearing 1-2 stiff setae distally and typically 1-2 additional stiff setae along the margin, basis anterior and posterior margin each with 1-2 simple setae.

**Figure 8.**
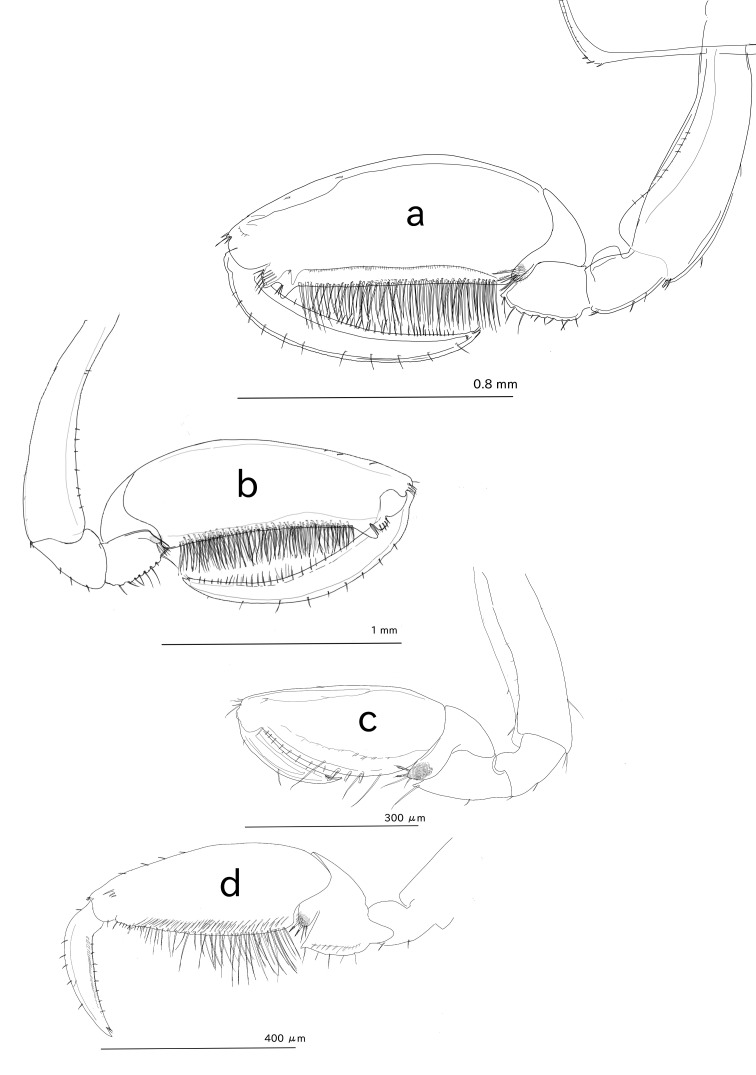
Gnathopod 2 of *Stenothoe gallensis*: **a, b** hyperadult male; **c** female; **d** younger male (subadult). Scale bar = length of palmar margin of Gn2.

Gnathopod 2 - Gn2: Gn2 (Fig. 8a and b) propodus mean length 1.10 ± 0.15 mm, about 2.8x propodus width (mean propodus width: 0.39 ± 0.05 mm); propodus palmar margin densely setose, with 6-8 of long simple setae, posterodistal end of palmar margin in males also with a triangular tooth and a blunt, crenulated projection with 4-6 short setae and two small setae inserted between the tooth and the projection; propodus palmar margin adorned with setae that reach an average length of 0.16 ± 0.03 mm, approximately 0.4x propodus width. Dactylus generally reaches the proximal end of propodus, with mean length 0.95 ± 0.17 mm, but occasionally shorter (Suppl. material 1, Figs S1 and S2). Dactylus inner margin in hyperadults is adorned with a row of short, simple setae, with a mean length of 55 **±** 13 μm (0.3 - 0.4x length of propodus palmar margin setae) as well as two simple setae inserted at the tip of dactylus; outer margin with scattered short setae. Carpus triangular with a patch of short, simple setae and a crown of both long comb-like stiff and simple setae (two stiff and 4-5 simple setae), merus crenulated with a row of simple setae present between crenulations; basis smooth without crenulations.

Peraeopods: Peraeopods 3-5 (Fig. 7IIc) with a long basis, less wide than the basis of peraeopods 6-7. Peraeopods 6-7 (Fig. 7IId) basis normally expanded, merus posterodistal lobe produced, not reaching more than half of the length of the carpus distal margin. Ratio of basis: ischium: merus (including posterodistal lobe): carpus: propodus: dactylus of P7 is 2.1:0.5:1.7:1.2:2:0.8.

Pleon: Pleopods with two distal coupling spines. Posterior angle of epimeral plate 3 distally acute.

**Figure 9.**
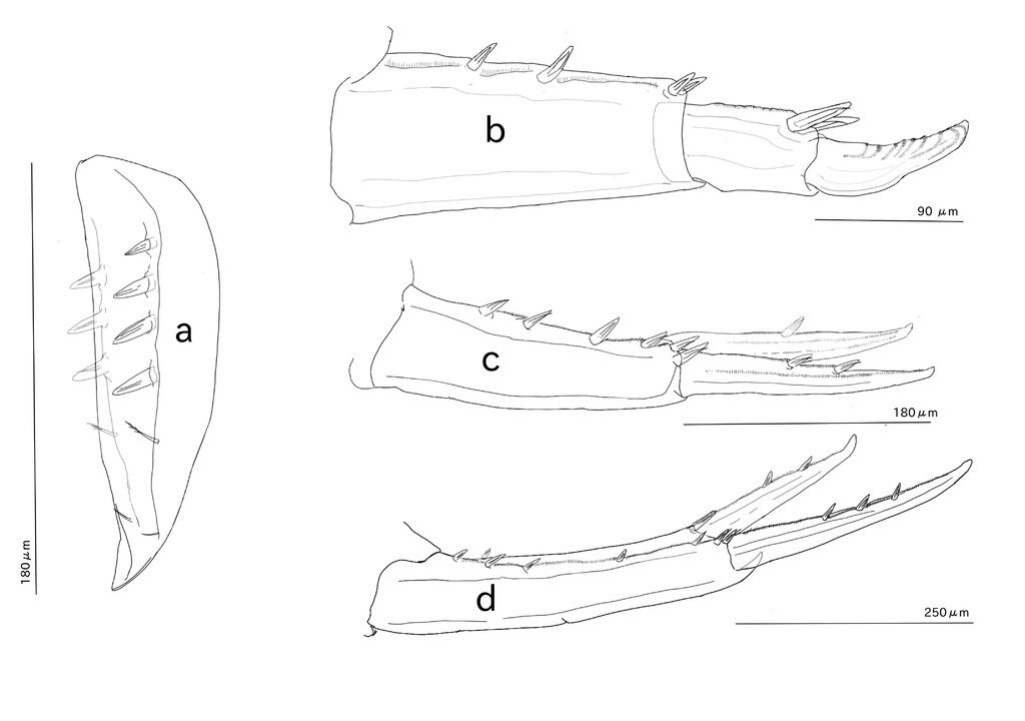
*Stenothoe gallensis*, line drawings of telson and uropods 1-3 of the hyperadult male (telson with three and four robust setae on each side of the proximal margin, peduncle of uropod 3 with two marginal robust setae and uropod 1 with distoventral spur).

**Figure 10.**
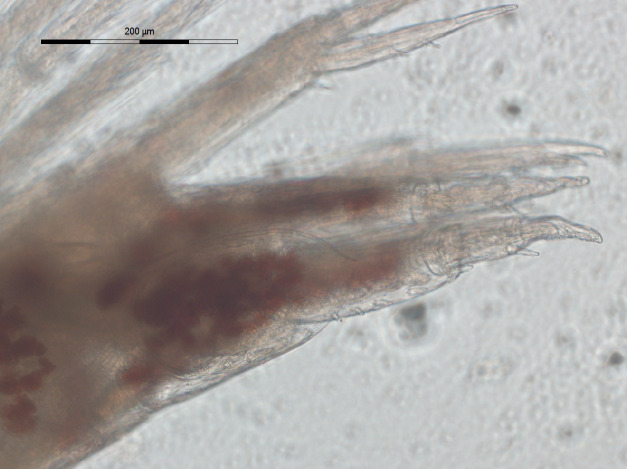
*Stenothoe gallensis*, shape of telson and armature of uropod 3 of a hyperadult male specimen. Telson with four robust setae on one side and peduncle of uropod 3 with four robust setae.

Telson: Telson ovate, lateral margins evenly convex; tapering distally to a narrow apex (Fig. 9a and Fig. 10); armature of telson of males of all maturity stages with 2-5 robust setae (10-30 μm) on each side of the dorsal margin, located anteromedially, but hyperadults with 3-4 pairs. Two pairs of fine setae on the posterior part of the telson, with the most distal pair shorter than the other.

**Figure 11.**
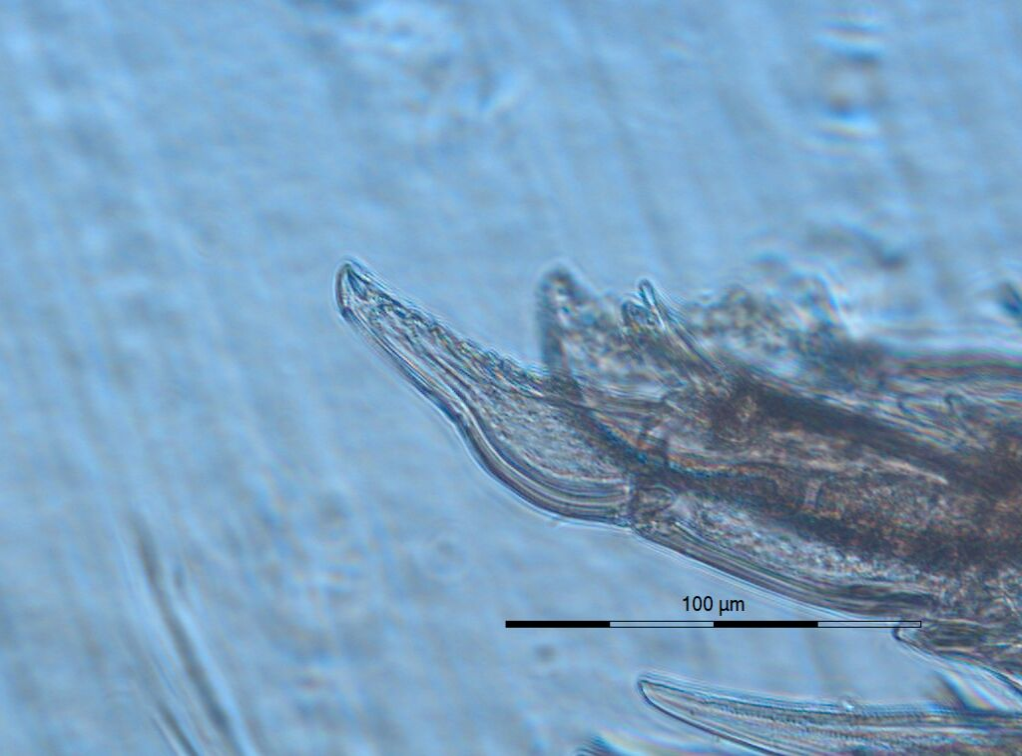
*Stenothoe gallensis*, detail of the crenulations on second article of a hyperadult male uropod 3.

Uropod 3- U3: U3 (Fig. 3b, Fig. 9b and Fig. 11) peduncle with 2-4 dorsal unpaired robust setae on outer margin and a pair of distal robust setae. Article 1 without marginal robust setae, but with a pair of distal robust setae, that are typically a little longer (30-40 μm) than the distal robust setae of the peduncle (20-30 μm). Article 2 distinctly rugose, markedly thicker proximally than distally; apex weakly recurved, terminating in a weakly pointed tip. The crenulations of the second article of U3 are adorned with tiny setae (Fig. 3b and Fig. 11). The approximate ratio of U3 peduncle: article 1: article 2 is 1:0.5:0.5. However, article 2 is typically slightly longer than article 1: 1:0.47 (± 0.056): 0.52 (± 0.064).

Uropod 2 - U2: Peduncle of U2 (Fig. 9c) without sharp ventral tooth; peduncle with 5-8 robust setae in total, 2 distal ones, inner and outer ramus subequal, outer ramus with 2-3 robust setae and inner ramus with 1-2, rami equal in length.

Uropod 1 - U1: Peduncle of U1 (Fig. 9d) with a sharp distoventral tooth between the rami; typically with 4-5 robust setae on upper margin and 2-3 robust setae tightly clustered together distally, 7-10 in total; outer ramus longer than inner, frequently with 3-4 robust setae on upper margin, inner ramus with 1-2. U1 usually reaches the end of U3.

##### Females (sexually dimorphic characters)

Female (Fig. 4) with TL of 5.06 ± 0.78 mm; largest individual 6.5 mm.

Antennae: Fig. 4d. Mean AL 1.35 ± 0.19; length about 0.4x body length. A1 ratio of peduncular articles 1-3 is 1:0.7:0.5; flagellum typically with 18 articles; A2 ratio of peduncular articles 4-5 is 1:1, with article 3 being 0.5x article 4; flagellum typically with 16-17 articles. Otherwise as in male.

Body: Mean BL 3.70 ± 0.63 mm; mean BW of 1.06 ± 0.19 mm.

Gnathopod 1 - Gn1: Gn1 (Fig. 7IIb) propodus relatively shorter than male Gn1; carpus triangular; merus shorter than male, weakly expanded; basis with several long setae.

Gnathopod 2 - Gn2: Mean Gn2 (Fig. 8c) propodus length of 0.37 ± 0.09 mm; mean propodus width: 0.17 ± 0.04 mm; mean dactylus length: 0.24 ± 0.06 mm; palmar margin subovate smooth, without teeth and with a few (3-4) long setae and 3-5 robust setae; inner margin with a few minute, scattered setae; carpus triangular with two comb-like stiff setae, one long simple seta and a patch of short setae; merus smooth, without crenulations.

**Figure 12.**
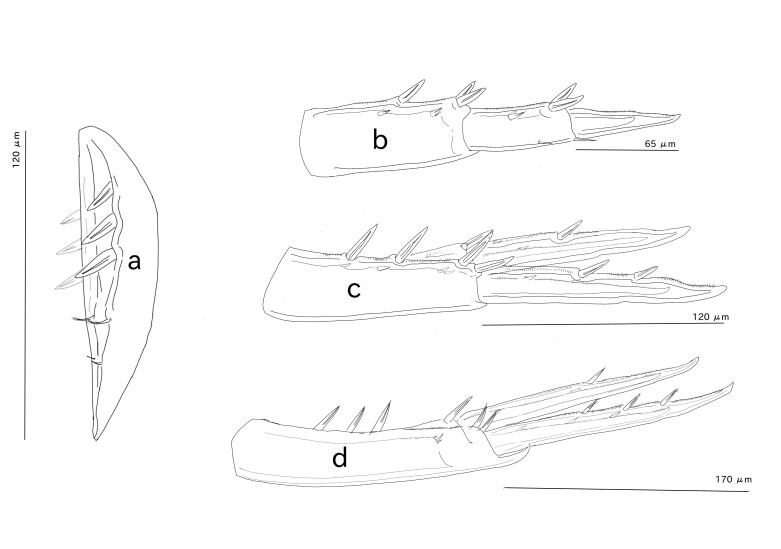
*Stenothoe gallensis*, line drawings of telson and uropods 1-3 of the female (telson with three and four robust setae on each side of the proximal margin, peduncle of uropod 3 with two marginal robust setae and uropod 1 with distoventral spur).

Telson: Telson (Fig. 12a) with 2-5 anteromedial robust setae on each side of the dorsal margin, usually 2-3.

Uropod 3 - U3: Uropod 3 (Fig. 12b) peduncle with 1-3 robust setae on outer margin, more frequently 1-2, as well as a pair of distal robust setae; art 1 with a pair of distal robust setae; art 2 straight, smooth, without crenulations. The approximate ratio of U3 peduncle: article 1: article 2 is 1:0.6:0.7, but this character can be variable.

Uropods 1-2 - U1, U2: Distroventral spur on U1 present (Fig. 12c and d).

##### Sub-adults/ Young males

Fig. 8d

Mean TL of 5.10 ± 0.92 mm.

Gnathopod 2 - Gn2: Gn 2 (Fig. 8d) propodus palmar and posterior margin with long, simple setae and small serrations along the length; dactylus reaching up to ¾ of posterior margin of propodus; inner and outer margin with scattered short setae; carpus triangular, posterodistal corner with long comb-like and simple setae; merus smooth, without crenulations and a row of simple setae.

In younger, smaller individuals the posterior margin is smooth, without serrations, like the gnathopod 2 of the female.

#### Diagnosis

**Regional**. Hyperadult male Gn2 palmar margin with numerous long setae (mean length: 0.16 ± 0.03 mm); posterodistal end with a prominent triangular tooth and a blunt, crenulated projection. Gn2 dactylus with short setae on inner margin. Gn2 merus posterior margin crenulate. Telson ovate, lateral margins evenly convex; tapering distally to a narrow apex with 2-5 robust setae on each side of the proximal margin, most commonly 3-4 and 2 pairs of fine setae on distal part of telson. Male uropod 3 peduncle with 2-4 unpaired marginal robust setae and a pair of distal robust setae, article 1 with two distal robust setae and article 2 strongly sculptured, markedly broader proximally than distally, weakly recurved with a pointed tip. The approximate ratio of U3 peduncle: article 1: article 2 is 1:0.5:0.5, with article 2 slightly longer than article 1. Right mandible incisor process typically with five teeth, 5^th^ tooth usually with two distal cusps; lacinia mobilis narrow, finely serrated. Left mandible incisor process and lacinia mobilis broad, minutely multidenticulate. Maxilla I palp distal article subequal in width to proximal article, but narrowing towards the apex. Plates of maxilla II separated; outer plate subequal in width, but 1.2-1.5x length of inner plate. Maxilliped palp 4-articulate; ischium (basal article of endopod) longer (1.4x) than palp articles 1 and 2 combined, with a pointed distolateral edge; inner plate of maxillipeds reduced; with two apical setae; length of inner plate 0.2x length of maxilliped palp ischium.

#### Statistical analysis of the morphometric variables

Regarding morphometric analyses, the Spearman rank correlations (Table 1) revealed significant positive correlations for all pairs of variables, with the exception of total body length and setae of Gn2 palmar margin for females. This highlights that diagnostic characters that are used for identification of members of the *S. gallensis* complex (i.e. length of Gn2 dactylus in proportion to Gn2 propodus, robust setae of telson and U3) are correlated with the size of the specimens, suggesting that the morphological variability presented in those characters is size related and, therefore, comparisons between descriptions should be treated with caution.

**Figure 13.**
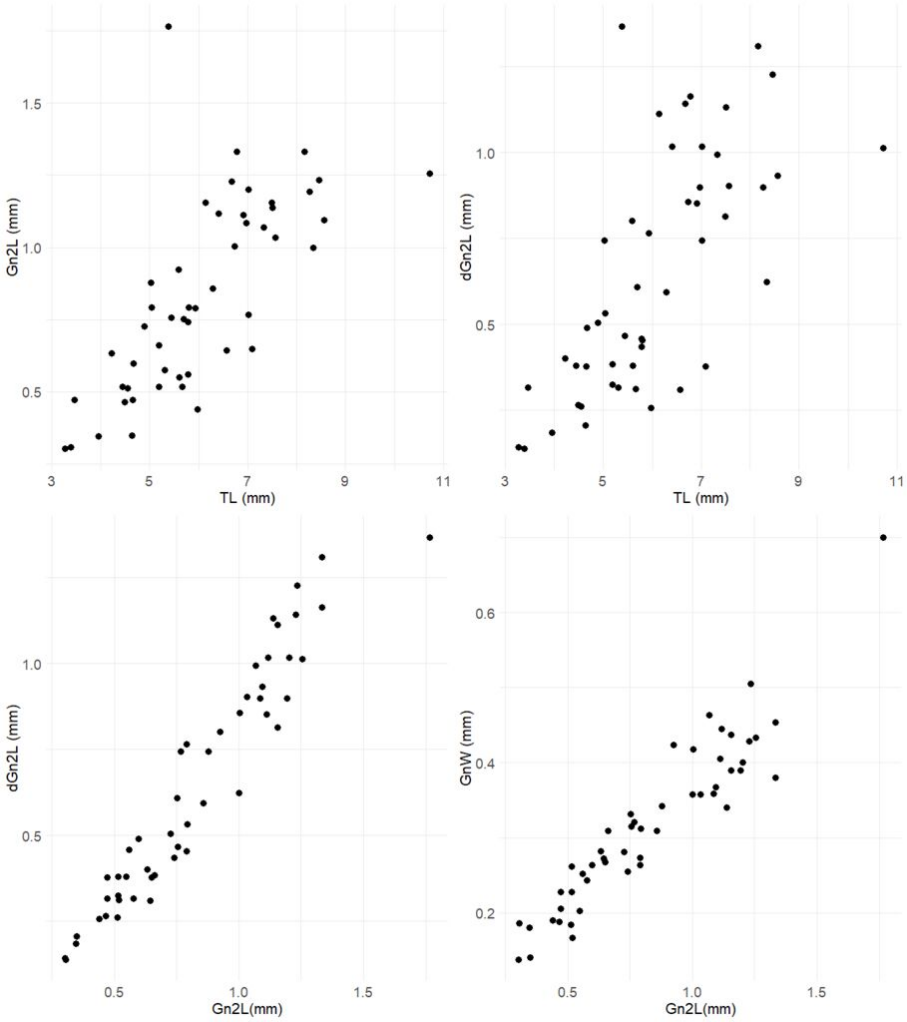
Scatter plots for visualisation of allometric relationships of male Gn2 propodus (Gn2L) and dactylus (dGn2L) length with total body length (TL), between propodus and dactylus and between width (Gn2W) and length (Gn2L) of propodus.

The scatter plots visualise the allometric relationships of male Gn2 (Fig. 13), with size of Gn2 propodus and dactylus increasing with specimen size.

The Mann-Whitney U test (Table 2) revealed significant variability (p-value < 0.05) between different maturity stages (hyperadults and younger individuals) for all examined morphometric variables. The character of dsGn2L was not included in the analysis since the row of inner setae of Gn2 dactylus could only be measured on hyperadults.

**Figure 14.**
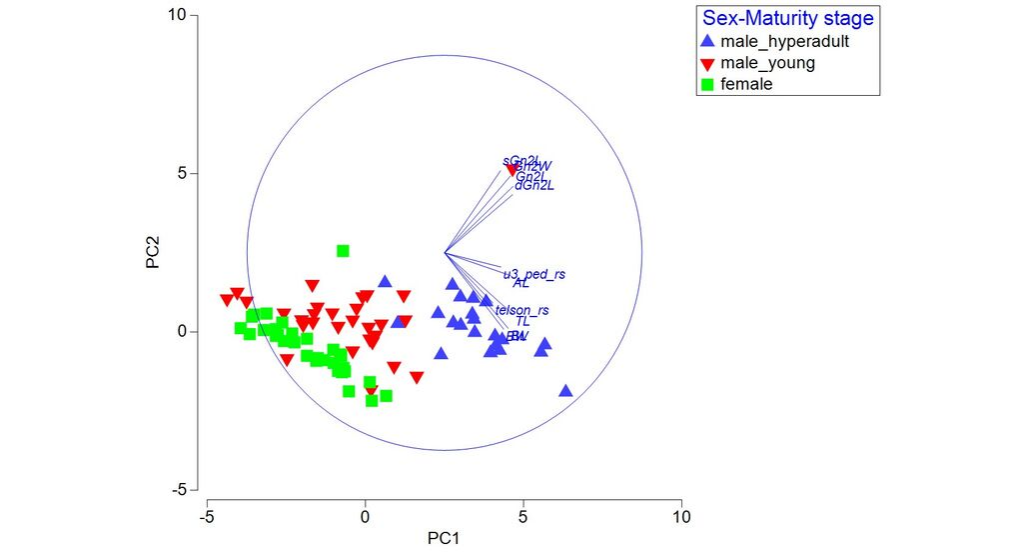
Principal Component Analysis (PCA) of morphometric variables between the different maturity stages and sexes (hyperadult males, younger male individuals, females).

The PCA (Fig. 14) revealed distinct clustering amongst male hyperadults, younger male individuals and females. PC1 accounted for 68.7% of the total variation, while PC2 explained 11.9%. Along PC1, hyperadult males were separated from females, with younger male individuals overlapping partially with both groups, but mostly with females, suggesting the incomplete development of secondary sexual traits. This pattern indicates that the primary axis of variation reflects sexual dimorphism, with a gradient corresponding to the progressive development of male-specific morphometric traits.

#### Remarks

The *Stenothoe* specimens examined during the present study exhibited several significant morphological similarities with *S. irakiensis*, including: (a) the configuration of the body; (b) the mouthparts; (c) the Gn1 and Gn2 morphology and dentition, and; (d) the armature of the telson and the male uropod. Concurrently, considerable variation was observed within the examined population, including amongst hyperadult males, in those characters having traditionally been accepted and used as diagnostic. Characters that present intraspecific morphological variation include: (a) the relative length of Gn2 dactylus (reaching or not reaching proximal end of palmar margin); (b) number of marginal robust setae on peduncle and rami of uropods 1-3; (c) armature of telson; (d) ratio of uropod 3 peduncle: article 1: article 2 (average ratio ± standard deviation: 1:0.47(± 0.056): 0.52 (± 0.064)); and (e) shape of the palmar margin of male gnathopod 2 (convex or concave, weakly, moderately, strongly). Nonetheless, the most important differences between species of the *S. gallensis* complex (and closely- related taxa, i.e. *S. lowryi* and *Stenothoe ogumi* Alves, Neves & Johnsson, 2020), based on currently available diagnostic characters, are highlighted in the comparative table of species provided in the supplementary material (Suppl. material 2 -Table S1).

Comparison with the original description of *S. gallensis* and *S. irakiensis* is particularly informative. In Walker’s (1904) description of *S. gallensis*, the male U3 peduncle is described as longer than the other two articles and with 5-6 robust setae on the upper margin, whereas the armature of article 1 is not explicitly described and appears to be illustrated with a single distal robust seta. On the other hand, Salman’s (1985) description of *S. irakiensis* shows 2-3 unpaired robust setae on the upper margin of the male U3 peduncle, in addition to a distal pair of robust setae, as well as two distal robust setae on article 1 and a strongly sculptured article 2. In these respects, the specimens collected from Greece agree more closely with *S. irakiensis* than with Walker’s brief original account of *S. gallensis*. It should be noted though that Salman (1985) reported greater variation of 7–24 segments in the A1 flagellum and 12–22 in the A2 flagellum, and that, according to Salman’s description, the accessory flagellum is minute with a single seta, but was not illustrated and could have been an erroneous observation, since this is also a character absent in all descriptions of the other members of the complex. Conversely, the original description of *S. gallensis* is limited in diagnostic detail, and some features that now appear taxonomically informative may simply have gone unreported. Accordingly, the available evidence does not permit a definitive separation between the two nominal taxa on morphology alone, but it does call for renewed scrutiny of their current synonymy.

The re-description of *S. gallensis* by Krapp-Schickel (2015) further complicates the issue. In that account, based on material from Dar es Salaam (Tanzania) rather than the type locality in Sri Lanka, the male U3 peduncle bears 5-6 robust setae, ramus article 1 has a single distal robust seta and article 2 is weakly sculptured and bent upwards medially. The material examined from Greece differs from this interpretation in showing two robust setae at the distal end of the first article, and a more distinctly sculptured second article. Krapp-Schickel (2015) also asserts that the U3 ratio of the peduncle and ramus is subequal for *S. gallensis*, a character which we found to be variable within the specimens from Greece, but with the second article of the U3 ramus frequently being longer than article 1. While these differences do not by themselves demonstrate that the material examined in this study represents a distinct species, they do show that the concept of *S. gallensis* currently in use is broader and less stable than is desirable for reliable diagnosis.

Walker’s statement that the mouthparts of *S. gallensis* are “as in *S. marina*” (Walker 1904) is also difficult to evaluate. Comparison with the illustrated account of *S. marina* (Bate 1857), as presented by Sars (Sars 1895, Plate 80) suggested broad similarity, but also notable differences in the relative size of the maxilliped inner plates. Compared with the mouthpart morphology of *S. irakiensis* and LeCroy’s diagnosis of *S. gallensis* (Lecroy 2011), the maxilliped outer plate ischium is illustrated as subequal in length to the combined first and second palp articles, rather than exceeding them, and the inner plate is less reduced than in *S. irakiensis*, in which it is depicted as approximately half the length of the outer plate ischium. Furthermore, variations in the morphology of the maxilla II plates are evident, characterised by an outer plate that is smaller in size relative to the inner plate, a distinction from the larger size of the outer article in *S. irakiensis*. However, due to the omission of mouthparts in Walker's illustrations of *S. gallensis*, direct comparison of with the topotypic *S. gallensis* is impeded. Additionally, in the preliminary description, U1 rami are described and illustrated with a single robust seta in the middle of the inner ramus, and 2-3 robust setae in the middle of the outer ramus. U1 and U2 peduncles are not described, but they are both illustrated with five robust setae, spread out in the upper margin of U1 peduncle and more tightly clustered together on the peduncle of U2.

Other illustrated descriptions of *S. gallensis* from the Indian Ocean include:

1. The description by Monod (1937) from the Suez Canal with a characteristic shape of male U3 art 2, which resembles *S. himyara* and *S. clavetta*, both described by Krapp-Schickel 2015. The second article of the male uropod 3 in *S. clavetta* is described as “being rounded and thickened proximally and abruptly narrowed in about half of the length, with the distal half about one third of the width, strongly sculptured and finger-like rounded ending.” (Krapp-Schickel 2015, figs. 12–13). Similarly, in *S. himyara*, the second article is described as having a “very peculiar shape being circularly rounded proximally and abruptly narrowed in about half of the length, with the distal half of about one third of the width, strongly sculptured and thumb-like rounded ending” (Krapp-Schickel 2015, fig. 17). However, *S. himyara* likely represents an immature male (see also Suppl. material 2), further complicating the comparison. Consequently, the morphology of the male gnathopod 2 (Gn2) cannot be reliably assessed. In the differential diagnosis between *S. clavetta* and *S. himyara*, Krapp-Schickel (2015) notes differences in body size (1.5–2.5 mm in *S. himyara*), the characteristic serration of the hyperadult male Gn2 (present in *S. clavetta*, absent in *S. himyara*), and relative antennal length (subequal in *S. clavetta*). However, body size in this range is also typical of immature males, and reduced or absent serration of the male Gn2, as well as variation in antennal proportions, likely indicates an ontogenetic stage rather than species-level differences (see also Discussion). In contrast, the male gnathopod 2 illustrated by Monod (1937) clearly exhibits the morphology characteristic of hyperadult males of the *S. gallensis* complex. Although we do not consider *S. clavetta* and *S. himyara* to be conspecific, given the substantial geographic separation between their type localities, the type locality of *S. himyara* (Port Sudan, Red Sea) is geographically closer to the locality from which Monod’s material was collected. This raises the possibility that Monod’s specimens may in fact belong to *S. himyara*. Nevertheless, the limited original descriptions and accompanying illustrations of all three species are insufficient to allow a conclusive taxonomic determination at present;
2. Nayar’s description (Nayar 1959) from Madras Harbour, which shows partial agreement with *S. irakiensis*, particularly in some mouthpart characters (notably the right mandible and maxilla 1) and in the armature of uropod 3. Nayar also noted that article 2 of uropod 3 is bent upwards medially (pl. V, fig. 16), a feature consistent with *S. irakiensis*. However, the description neither mentions nor illustrates the crenulation of uropod 3 and the male gnathopod 2 is depicted with short setae along the palmar margin, differing from *S. irakiensis*;
3. Ledoyer’s material (Ledoyer 1986) from the Glorious Islands, which differs more substantially with two robust setae on each side of the telson (as compared to the three pairs of Walker’s original description), as well as male U3 peduncle a little shorter than the articles of ramus, which art 2 referred to as “occasionally crenulated”, may simply mean that part of the examined material might belong to the *S. valida* species complex (Lecroy 2011). It also depicts male gnathopod 1 with a short propodus, equal to the length of carpus and merus. U1 peduncle, U2 peduncle and rami are also depicted with fewer robust setae, though the exact number is not detailed.

Taken together, these Indian Ocean and adjacent region records suggest that historical usage of the name *S. gallensis* has probably encompassed more than one morphotype. Many extra-type records assigned to *S. gallensis* are, therefore, not directly comparable at the level of detail required for secure species identification. Outside the Indian Ocean, the same pattern of uncertainty is evident. Illustrated records of *S. gallensis* include:

1. The description by Reid (1951) from West Africa (Banjul, Gambia), in which he notes that the records had a degree of uncertainty, partly attributed to the ambiguity of earlier descriptions. This description, similar to other illustrated accounts of *Stenothoe*, was incomplete as it did not describe several identification characters (e.g. male U3 peduncle armature). Krapp-Schickel (2015) described a new species from Senegal, *Stenothoe senegalensis* and compared the material of the new species with Reid’s original description, considering it referable to this species. However, given the incomplete nature of Reid’s description, it is difficult to ascertain the validity of this assessment;
2. Illustrated description of material from the Yellow Sea (Ren 1992, Ren and Sha 2015), which reports a telson with 3-4 robust setae on each side and a crenulated male U3 with marginal robust setae on the peduncle. Some of the mouthparts are also depicted (incisor, right mandible). However, the specimen that is illustrated appears to be a younger individual, since the dactylus of male Gn2 is depicted as relatively short and without setae on the inner margin and the male size also mentioned in the description is 4.5 mm for males.
3. LeCroy’s (2011) Florida material, which is especially noteworthy because the armature of the male uropods resembles Salman’s *S. irakiensis* closely. The tip of peduncle U1 is illustrated with three tightly clustered distal robust setae and a distoventral spur, a detail only recorded by Salman (1985). The Gn1 is described with the posterior margin of propodus straight to slightly concave and subequal to the palmar margin, but the shape is not illustrated. Gn2 is similar to both Salman’s and Walker’s descriptions. This illustration is also lacking the shape of mouthparts, with the exception of maxillipeds and upper lip;
4. A few illustrated descriptions of *S. gallensis*, provided by Azman (2023), are for different species. For example, *Stenothoe* specimens Shoemaker (1935) from Puerto Rico were attributed to *S. crenulata* by Krapp-Schickel (2015), which was described by Chevreux (1907) from French Polynesia. Regarding this species, Krapp-Schickel (2015) also studied specimens from Barbados (Caribbean Sea), Bali (Indonesia) and Curaçao (Caribbean Sea) and assigned all of this material to *S. crenulata*, providing an accompanying description and illustrations. However, the description also incorporated material from the Pacific Ocean attributed to *Stenothoe cf. crenulata* and these specimens differ in several respects from those recorded from the Caribbean Sea (see Suppl. material 2 - Table S1). In addition to that, the material from Puerto Rico described by Shoemaker is different from the material described originally by Chevreux. Some of these differences, as noted by Shoemaker, include the second coxa (coxa 2) being broader and less evenly rounded than in Chevreux’s specimens, the rudimentary mandibular palp bearing a single seta instead of two and the posterior margins of gnathopod 1 being parallel rather than divergent. An additional difference, not previously mentioned, is that, in Shoemaker’s specimens, the first article of uropod 3 is distinctly shorter than the peduncle, whereas in Chevreux’s specimens, it is longer. Therefore, the proposed broad geographic distribution may not be strongly substantiated;
5. Azman (2023) also includes descriptions and illustrations from *Stenothoe* from France (Chevreux and Fage 1925) and England (Lincoln 1979). Both of these descriptions have been attributed to *Stenothoe cattai* by Krapp-Schickel (2015). The case of *S. cattai* is also not straightforward. As discussed by Krapp-Schickel, Stebbing’s original description (Stebbing 1906) is extremely limited, lacks illustrations and is based largely on Catta’s description (Catta 1876) of *Probolium polyprion*, which, in contrast, includes a detailed description and illustrations. Catta himself did not detect differences between *P. polyprion* and *Probolium megacheles*, the latter being a synonym of *S. valida* (Krapp-Schickel 1974). Nevertheless, *P. polyprion* cannot be referred to *S. valida*, as it differs in having the propodus of gnathopod 1 longer than the carpus and merus and in the posterodistal lobes of the merus of pereopods 6 and 7 not reaching the distal margin of the carpus (see also Remarks on *S. valida* below). It is also noteworthy that Catta’s specimens originated from the port of Marseille, collected from ship hulls of a vessel recently arrived from India. Krapp-Schickel (1976) illustrated hyperadult specimens from France and Italy, but also from outside the Mediterranean, including Bermuda (Krapp-Schickel 2015). Even amongst the Mediterranean material from France and Italy, differences in the illustrations are apparent in the setation length of gnathopod 2 (see also Suppl. material 2 - Table S1), raising some uncertainty as to whether all Mediterranean material examined by the author represents a single species. Nevertheless, the diagnostic characters of the male uropod 3 armature and the relative length of the gnathopod 1 propodus — features also reported in the material described by Chevreux and Fage (1925) — support the distinction of this species from *S. gallensis*. Catta’s material shows closer resemblance to the specimens examined in this study, particularly in the morphology of the mouthparts and uropod 3; however, differences, such as the presence of a series of marginal telson setules inserted in notches laterally and the more rectangular shape of the gnathopod 1 propodus with parallel rather than slightly divergent margins, prevent reaching a more definitive conclusion;
6. One of the more recent records of the species for the Mediterranean Sea, which was briefly described and illustrated by Zakhama-Sraieb and Charfi-Cheikhrouha (2010) from the Tunisian coasts. Based on the taxonomic characters they provided, the material they examined does not appear to correspond to those of *S. cattai*. However, although the male U3 and telson armature were not described, the illustrations depict the U3 peduncle with two marginal unpaired robust setae and a distal robust seta instead of two on the peduncle, two distal robust setae on art 1, while the telson is depicted with two robust setae on one side and three on the other. U1 peduncle is also depicted with only two marginal robust setae. Aside from the shape of Gn1 and Gn2, other important diagnostic characters like crenulations of U3 art 2 and the shape of mouthparts were not described. This, combined with illustrations of U3 art 2, which are not representative of *S. gallensis*, leads us to doubt the validity of the record.

The specimens that were identified in this study also resemble *Stenothoe senegalensis* in the shape of the male Gn2 palm, which has long setae and a distally narrow U-shaped excavation, followed by a prominent tooth, as well as the shape of the U3, with art 2 of the ramus being proximally thicker than distally, strongly rugose with a recurved tip. However, in the description of *S. senegalensis* (Krapp-Schickel 2015), the tip of art 2 is acutely uncinate, whereas in these specimens, the tip is more blunt and not as recurved. There are also some other differences, which include: (1) the shape of the mandibles, which does not match the depiction in the figures of the description of *S. senegalensis* (refer to Suppl. material 2 and Krapp-Schickel (2015), fig. 19) and (2) the lack of a notch on the anterodistal corner of coxa 4. This species also resembles *S. irinae* in the shape of the palmar margin of male Gn2 propodus, but can be separated from it by the presence of at least two unpaired robust setae and a pair of distal ones on the peduncle of uropod 3 instead of two dorsal robust setae in *S. irinae* (one unpaired and one distal). Additionally, *S. irinae* is armed with a very large tooth of Gn2 and, regarding the mouthparts, the plates of maxilla II are fused, whereas in the specimens of this study, they are separated.

*Stenothoe lowryi* Azman 2023, the most recently described member of the *Stenothoe* genus, is remarkably similar to the specimens collected by the current study and to Salman’s description of *S. irakiensis*, although not stated to belong to the *S. gallensis* species complex. This is attributed to the second article of the male uropod 3, which is not rugose and clearly differentiates this species from other members of the species complex. Azman also mentions as differences from *S. irakiensis* uropod 2 rami with only one robust seta on medial margin, while *S. irakiensis* hyperadults frequently have uropod 2 outer ramus with three robust setae and inner ramus with one marginal robust seta and occasionally a single robust seta on the surface. Azman (2023) also notes that *S. lowryi* differs from *S. irakiensis* in having uropod 3 article 2 distinctly longer than article 1 (instead of slightly longer) and male gnathopod 2 propodus with palmar margin moderately convex instead of moderately concave. These characters are to be regarded with caution as they may not serve as reliable diagnostic features, given the observed variation occurring in the specimens examined during this study.

Outside of the *S. gallensis* species complex, *S. valida* is a very closely morphologically related species (Dana 1853). Amongst the *Stenothoe* populations of this study, a few male individuals belong to *S. valida*, which has also been suggested as a species complex (Krapp-Schickel 2015, Marin and Sinelnikov 2018). However, these individuals can be differentiated from members of the *S. gallensis* complex by the straight article 2 of male uropod 3, the greatly expanded merus (wider and longer) of peraeopods P6 and P7, with produced posterodistal lobe close to distal end of carpus and the absence of a distroventral spur between U1 rami. Mouthparts are also different since individuals that belong to *S. valida* have an upper lip with unequal lobes and the ischium of maxillipeds is subequal or shorter than the total length of palp articles 1 and 2. Males of *S. valida* have a straight margin of Gn2 merus instead of crenulated, but subadults of *S. gallensis* have less pronounced crenulations and a straight margin in immature individuals, similar to the *S. gallensis* female Gn2. Females of the two species are easily confused with members of the *S. gallensis* species complex since they are similar, but *S. valida* females can be differentiated by the following: larger and pigmented eyes, different body size and shape of Gn2 with 1-3 distal palmar teeth on the Gn2 propodus palmar margin (Dana 1852, Lecroy 2011, Paz-Ríos et al. 2013, Krapp-Schickel 2015). In light of these new data from the present study, the species concept of the *S. valida* complex is also being challenged. Such an example is represented by *Stenothoe ogumi* Alves, Neves & Johnsson, 2020, which was regarded by its authors as closely related to both *S. valida* and *S. gallensis*, since it exhibits characters traditionally associated with both complexes. Specifically, the maxilliped ischium in *S. ogumi* is longer than palp articles 1–2 combined, a character regarded as typical of the *S. gallensis* complex by Lecroy (2011), who regarded the opposite character state (maxilliped ischium shorter than palp articles 1–2 combined) as diagnostic of *S. valida*. Nevertheless, Alves et al. (2020) considered *S. ogumi* to be allied to the *S. valida* complex, based primarily on the expanded merus of pereopods 5–7. However, according to their description and illustrations, these segments are not markedly elongated, with the posterior margin reaching only approximately half the length of the carpus. In addition, consistent with *S. valida*, no distoventral spur on uropod 1 was illustrated and the male gnathopod 2 possesses a smooth, uncrenulated merus (provided that the illustrated specimen is correctly interpreted as the hyperadult male, which may not be the case here, refer also to Suppl. material 2 - Table S1). Conversely, the male was illustrated with uropod 3 article 2 straight and smooth, but bearing a small subtriangular distal projection and three robust setae, whereas in *S. valida*, this article is entirely straight and lacks such a projection (see also Suppl. material 2 - Table S1). These observations suggest that many of the morphological characters traditionally used to distinguish members of the *S. valida* and *S. gallensis* complexes may be unreliable for species delimitation. Consequently, species hypotheses within the *S. valida*/*gallensis* complex will require molecular data to clarify species boundaries and resolve the taxonomy of the group.

**Figure 15.**
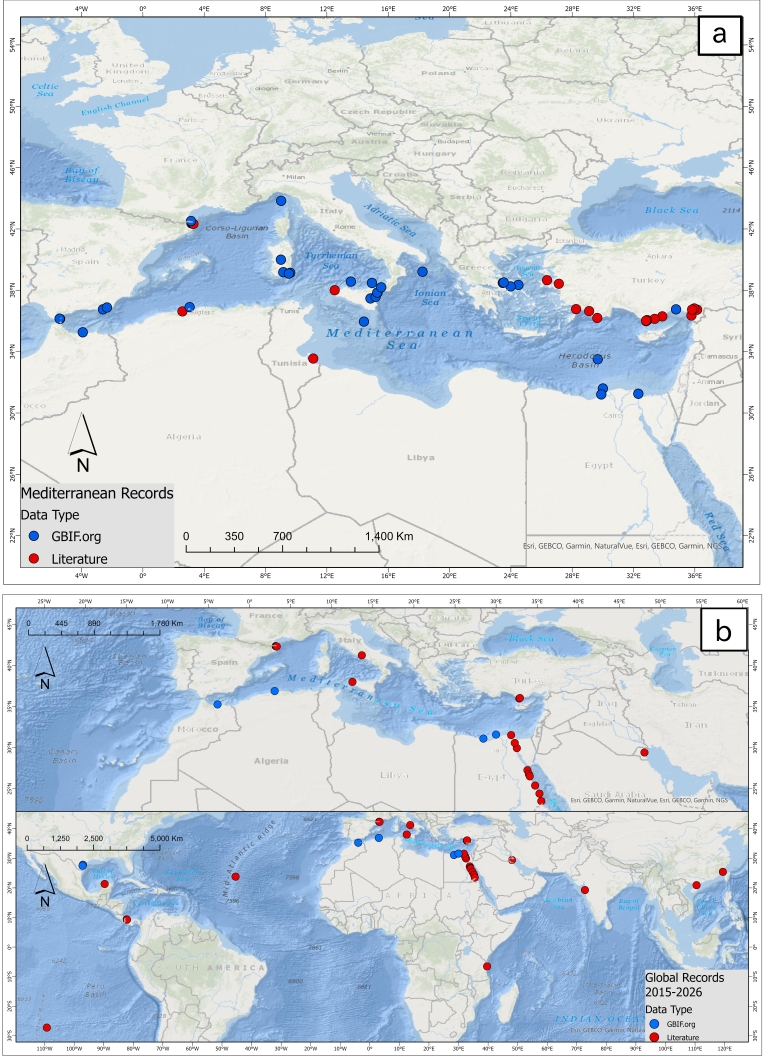
Maps showing (a) Mediterranean records of *Stenothoe gallensis* Walker, 1904 and (b) Mediterranean and global records of *Stenothoe gallensis* Walker, 1904 from 2015-2026, following the revision by Krapp-Schickel (2015).

Historical Mediterranean records of the species are presented in Fig. 15a and include records up to 2026. Although these records may represent different species, such as *S. cattai*, the earliest records originate from the eastern Mediterranean, including the Levantine and Aegean Seas (see also Suppl. material 4). For the updated global dataset (2015-2026) (Fig. 15b), most records are concentrated in the Levantine Sea and the Suez Canal, which may suggest the introduction pathway via the Suez Canal. Genetic evidence would be required to clarify the introduction pathway and dispersal patterns.

## Discussion

The results of the quantitative morphometrical analysis carried out in the context of this study revealed that some key diagnostic characters used to distinguish species of the *S. gallensis* species complex are more variable than previously assumed. In particular, the number of robust setae on the telson, the dorsal unpaired robust setae of the peduncle and the relative proportions of male appendages present significant variation and depend on the maturity stage and the sex. Thus, the maturity stage of the male should, therefore, be considered explicitly in species diagnosis within the complex and it is recommended to use hyperadult individuals for identification and description, as, in some occasions, subadult males have even been mistakenly described or identified as separate species from the adults (Lecroy 2011). Such a case is exemplified by *S. dentirama* (Hirayama and Takeuchi (1993), type locality: Fukushima, Japan), whose type material likely represents an immature male. Even when a holotype appears morphologically distinctive, key male characters, such as gnathopod 2 shape, may not be fully developed, which can contribute to taxonomic uncertainty (refer also to Suppl. material 2). Consequently, one of the principal diagnostic characters distinguishing other species within the complex, later incorporated by Krapp-Schickel (2015) into her key as “Gn 2 propodus male and female similar, proximally rounded and distally continuously narrowing, hind margin smooth”, cannot be reliably assessed, thereby contributing to ongoing taxonomic uncertainty. Another similar example is the case of *S. himyara* (see Remarks). Furthermore, a more recent case is the *Stenothoe cf. tergestina* (Nebeski 1881) recorded from Black Sea (Grintsov 2024). The U3 article photographed by SEM is showing crenulations similar to the *S. gallensis* complex; however, the specimen clearly belongs to a subadult individual. Based on the shape of the second article of U3, which is different from both *S. gallensis* or *S. cattai*, perhaps this material belongs to a different, perhaps undescribed, species, but information only of the subadult is limiting.

Additionally, variation in diagnostic characters, such as the relative length of male Gn2 dactylus to propodus (reaching, not reaching or surpassing proximal end of propodus) and the length of setae on the inner margin on male Gn2 dactylus, can be observed even amongst hyperadult males. The allometric growth observed in male Gn2 may reflect ecological selection pressures associated with competition, resource access or mating success (Cherry et al. 2020, Premate et al. 2024, Danole et al. 2026); hyperadults have longer gnathopods that are also wider, with longer dactyli and more developed setae. This role, however, may differ between amphipod species. For example, a study for the amphipod *Ampithoe ramondi* showed a positive correlation of Gn2 with body size, which is likely linked to reproductive success, whereas the amphipod *Caprella acanthifera* exhibited limited sexual dimorphism, potentially reflecting the non-territorial resource use, broad diet and high mobility of the species (Danole et al. 2026). In members of the family Stenothoidae, the pronounced sexual dimorphism has also been suggested to be associated with mate-guarding behaviour (Conlan 1991). Furthermore, we tentatively assume that moulting cycle stages with the progressive retraction of the old cuticle of the appendage and the synthesis of a new cuticle are likely to be, at least partially, connected to the developmental variation presented in some characters, particularly in uropods, such as the number of marginal robust setae. This assumption comes from the discrepancy that was occasionally observed in the number of marginal robust setae of the old cuticle and the new cuticle (Fig. 3b) and since size variation that is age-related and increasing at each moult has been also previously recorded in amphipods (Davolos et al. 2018). Finally, small, immature males that have not yet developed a sexually differentiated gnathopod 2, characterised in subadult males by a longer, more defined propodus with serrations along the posterior margin, can nevertheless be distinguished from females by the rugose third article of the male uropod 3, although this can be more or less sculptured and we believe to be associated with size. In our study, *S. gallensis* hyperadult males were, on average, the largest individuals, whereas subadult males differed only slightly in body length from females. However, it should also be noted that, in a population dynamics study of *S. valida*, reduced male size was suggested to be associated with reduced male–male competition across different time periods (Lee and Park 2021), as high population density leads to increased sexual selection on male body size (Nahavandi et al. 2011). Since the key diagnostic characters that are used in the identification of members of the *S. gallensis* species complex present these size-dependent variations, the taxonomic distinction between species is rendered challenging as species descriptions are frequently based on the assignment of a holotype and perhaps a few paratypes.

Sexual dimorphism and ontogenetic variation are important considerations in species delimitation across many amphipod families, not only in Stenothoidae. In Mediterranean amphipods, sexual dimorphism is common and is often more pronounced in males. Prominent examples occur in several families of the infraorder Corophiida, including Corophiidae, Ischyroceridae, Aoridae, Photidae, Isaeidae, Ampithoidae and Caprellidae (Myers and Lowry 2003), although sexual dimorphism is also well documented in other families, for example, Gammaridae (Hume et al. 2005), Maeridae (Lowry and Hughes 2009) and Melitidae (Krapp-Schickel and Sket 2015), where males frequently exhibit distinctively enlarged or modified appendages. Consequently, the recognition of sexual dimorphism and ontogenetic stages is essential for accurate species descriptions and for avoiding misidentifications and taxonomic overestimation. For example, species of the genus *Jassa* have historically caused taxonomic confusion because a sexually dimorphic secondary sexual character develops only after the final moult, leading to pronounced differences amongst ontogenetic stages (Conlan 1989).

In the aquaculture facility located in Saronikos Gulf, amongst the amphipod species that were recorded to co-exist with *S. gallensis* were *Ericthonius brasiliensis* (Dana, 1853), *Jassa cf. slatteryi* Conlan, 1990, *Laticorophium baconi* (Shoemaker, 1934), *Elasmopus rapax* A. Costa, 1853, *Caprella dilatata* Kroyer, 1843 and *Caprella equillibra* Say, 1818. In the aquaculture facility located in Rhodes, the amphipod records associated with *S. gallensis* included *Elasmopus rapax* A. Costa, 1853, *Ampithoe bizseli* Özaydinli & Coleman, 2012, *Ericthonius brasiliensis* (Dana, 1853), *Jassa cf. slatteryi* Conlan, 1990, *Pseudoprotella phasma* (Montagu, 1804) and *Caprella equillibra* Say, 1818. A few specimens of *S. valida* were also found to co-exist with the populations of *S. gallensis*. The NIS species *L. baconi and J. slatteryi* have been previously recorded from marinas in Greece and are associated with fouling assemblages (Guerra-García et al. 2023, Chatzigeorgiou et al. 2026). Likewise, the presence of the cryptogenic species *E. rapax*, *E. brasiliensis, C. equillibra* and *C. dilatata* in the Mediterranean have beeen associated with fouling communities and can be found on ropes in Mediterranean off-coast aquaculture facilities (Fernandez-Gonzalez and Sanchez-Jerez 2017, Revanales et al. 2022, Calabrese et al. 2025, Özgen Çoban 2025). Additionally, the cryptogenic *A. bizseli* was originally described from İzmir Bay and has subsequently been recorded from Turkey, Tanzania, Cyprus and Spain and is also known to occur in fouling communities associated with aquaculture sites (Özaydinli and Coleman 2012, Arduini and Mancini 2025). Apart from *S. gallensis*, the non-indigenous, rapidly established *S. georgiana* was also identified in the present study, occurring at one of the three aquaculture locations examined (Aq2). Although this species was only recently reported from Greece, based on material collected during 2021–2022 (Chatzigeorgiou et al. 2026), its actual presence in the region may precede these records, implying that it has likely been under-reported. Furthermore, integrative molecular studies addressing its status as a neo-cosmopolitan species and its introduction pathways are still pending (Martínez-Laiz et al. 2020). The amphipods that were associated with the presence of *S. georgiana* were *E. rapax*, *J.* cf. *slatteryi, L. baconi, C. dilatata* and *C. equillibra*. Currently, only four records of *S. gallensis* are reported from Greece (GBIF 2026). Three of these originate from both northern and southern Evoikos Gulf and date from 1937. In our material, *S. gallensis* was absent from the northern Evoikos Gulf (station Aq2), where only *S. georgiana* was recorded. A more recent record (2012) of *S. gallensis* originates from the Aegean Sea (coast of northern Euboea). *S. gallensis* has also been included in regional checklists from the Aegean and Levantine Seas (Kocataş & Katağan 1978; Christodoulou et al. 2013; Çınar et al. 2017), whereas it is absent from records in the Ionian Sea (Katsanevakis et al. 2011). This distributional pattern likely reflects under-reporting from Greece rather than a true absence from parts of the region.

As many identifications of material from the Mediterranean prior to the re-description of *S. gallensis* by Krapp-Schickel (2015) relied on incomplete or inconsistent earlier descriptions, records published before 2015 should be treated with caution. Additionally, Evans et al. (2015) considered the records of *S. gallensis* from the Maltese Islands (Central Mediterranean) as “casual?”, as they relied only on the records by Fernandez-Gonzalez and Sanchez-Jerez (2013), which lacked description and information on specimen abundance, as well as corroboration from other records. After 2015, the records of *S. gallensis* in ecological studies are scarce. *S. gallensis* has even been suggested to be removed from inventories of alien species in Mediterranean waters (Evans and Schembri 2016). Pinedo et al. (2024) collected material in 2019 from shallow habitats (< 0.2 m depth, *Ellisolandia elongata* (J.Ellis & Solander) K.R.Hind & G.W.Saunders, 2013 turfs) in northern Catalonia (NW Mediterranean) and in high abundances. Although the basis of these records was not specified, we assume that the actual distribution of material referrable to the *S. gallensis* complex in the Mediterranean is likely to be underestimated. The most recent record from the Mediterranean Sea (Badalucco et al. 2026) is based on a brief description of specimens from the vicinity of Trapani (Sicily, Italy), which is accompanied by limited photographic material. Although the shape of U3, Gn1 and Gn2 visible in the photographs suggests that the specimens may be comparable to our material, other morphological details are not discussed further, which prevents a more thorough evaluation. From the type locality of *S. irakiensis* (Arabian Gulf), a more recent record of *S. gallensis* from Kuwait (Al-Yamani et al. 2019) includes photographs that closely match the original description of *S. irakiensis* (note that, although the photographs show a male uropod 3 with two distal robust setae on the first article, this feature was not illustrated in the accompanying drawings).

Up until now, most of the descriptions and illustrations for *S. gallensis* do not include illustrations and/or description of some details that we believe to be important. For many of them, for example, the mouthparts illustrations are lacking and even the more detailed descriptions only depict one pair of the mandibles. In some descriptions, like *S. gallensis* by Nayar (1959), mouthparts were not illustrated at all. Additionally, the most important diagnostic characters that have been considered up until now for the description of species (e.g. the armature of male uropods and, particularly, the shape and armature of U3) are often overlooked. Given the limited detail in the original description of *S. gallensis* by Walker (1904), there is an evident need to collect and re-examine material from the type locality in order to re-instate *S. gallensis*. Lack of detail in descriptions of characters and/or absence of topotypic material in the subsequent re-descriptions have also introduced uncertainty for all members of the complex. Furthermore, lack of knowledge in the plasticity of some characters restricts us from assessing the validity of their diagnostic accuracy. For example, in some *Metopa* species, which also belong in the Stenothoidae family, the defining character of the articulation of mandibular palp has been suggested to be plastic (Tandberg and Vader 2009). Since, similar characters are also used in identification of members in the *S. gallensis* species complex, such as whether maxilla II plates are fused or separated, examining plasticity may prove valuable for future studies. Nevertheless, the status of *S. irakiensis* as a junior synonym of *S. gallensis*, as well as that of *S. gallensis* remains somewhat ambiguous. The question that subsequently arises is what constitutes a member of the “*S. gallensis*” species complex? The shape of the second article of male uropod 3 is the most obvious character that separates *S. gallensis* from its remaining congenerics. However, this character varies between species, both in the shape of the curve (strongly or weakly upwards bent, not upwards bent, but medially widened or thickened etc.) as well as the serrations (more or less serrated). In the case of *S. lowryi*, serrations are absent, yet in all other characters, it matches the *S. gallensis* species group (see also Suppl. material 2). Re-examination of the morphological characters that comprise the diagnosis of *S. gallensis* will add valuable insight for morphological delimitation of species outside and within the complex.

This study highlighted that accounting for intraspecific morphological variation is essential for robust taxonomic studies, particularly for already established and/or potential species complexes, as it enables a better understanding of the distinction between true species-level differences and variation occurring within a single species. This approach would be especially effective when molecular data are available to support taxonomic hypotheses (Mamos et al. 2014, Hupało et al. 2020, Delić et al. 2025). While molecular correction of misidentifications and species delimitation is important, monitoring and biodiversity studies that rely on morphological descriptions for species identification often use only a few diagnostic characters instead of comprehensive statistical analyses of intraspecific morphological variation, making it challenging to differentiate between closely-related species. The limited use of such analyses in practice outside of species descriptions highlights the need for a wider application in species records and revisions (Pilgrim and Darling 2010). Careful evaluation of key meristic and/or linear diagnostic characters provides a quantitative framework for identifying consistent patterns and assessing the reliability of diagnostic traits used in species delineation. Especially in the case of invasive species, phenotypic variability plays a crucial role in facilitating their spread and successful establishment in new environments (Podwysocki et al. 2024), while also potentially obscuring taxonomic boundaries within species complexes. These challenges become even more pronounced when material originates from outside the type locality, as geographically separated populations may exhibit variation not recorded in the original description. Consequently, detailed re-descriptions of material attributed to a species *sensu lato* from non-type localities are important, as they provide a basis for direct comparisons between descriptions. Ultimately, integrating intraspecific morphological variation into taxonomic studies strengthens taxonomic accuracy and reduces the risk of misidentification or taxonomic inflation. Molecular investigation could provide further taxonomic clarification for species delimitation, validate previously established morphospecies within the complex and be valuable for determining phylogenetic relationships with the morphologically distinct members of the *S. gallensis* complex. Genetic material from the type locality of *S. gallensis* (Sri Lanka) is urgently needed to establish a reference framework for evaluating extra-type populations and testing whether currently used morphological characters reliably delimit species. Molecular data for this species remain scarce; only six nucleotide sequences are currently available in GenBank, none of which originates from the type locality. Therefore, sequences collected from the type locality would also serve as reference sequences for species delimitation when comparing sequences from other localities, thereby assessing whether the conventional taxonomic characters we have established for morphological delimitation of species are successful (Jörger and Schrödl 2013) or whether the re-instatement of additional taxonomic characters with a revision of the complex is necessary. Furthermore, application of population genetics and phylogeographic methods will aid in tracking dispersal pathways, as well as tracing the introduction routes of this species in the Mediterranean. A collaborative, integrative taxonomic study incorporating both morphological and molecular material from multiple type localities and contributed by various taxonomists would greatly facilitate the resolution of the species complex.

Finally, a formal revision of the *Stenothoe gallensis* complex is still lacking and a re-description of the nominal species, based on material from the type locality, is also required. The genus *Stenothoe* has historically received limited taxonomic attention, with only 31 first authors having described species to date. Amongst them, Krapp-Schickel is the most prolific (16 species), followed by Barnard (seven species) and Chevreux (five species). *Stenothoe ogumi*, is currently the most recently described species (WoRMS 2026). This limited taxonomic effort is reflected in the absence of a modern systematic revision of the genus. The most important contributions remain the cladistic analysis of Stenothoidae by Krapp-Schickel and Koenemann (2006) and the world identification keys provided by the same author in 2013 and 2015 (Krapp-Schickel 2013, Krapp-Schickel 2015).

Our review of literature on *S. gallensis* revealed inconsistencies amongst several nominal species within the complex. In light of these findings, together with the results of the present analysis of intraspecific morphological variation, we propose the following preliminary framework and recommendations for future taxonomic studies of the *S. gallensis* complex and the genus as a whole:

1. Descriptions based on hyperadult males. The examination and collection of hyperadult individuals should be considered essential in species descriptions and revisions. Particularly in *S. gallensis*, hyperadult males can be distinguished primarily by the size and morphology of gnathopod 2 (see Morphometrical and Statistical Analyses and Description sections). Comparative assessment of subadult males, hyperadult males and females is recommended, together with the reporting of size metrics for both sexes;
2. Documentation of morphological variation. Species descriptions and revisions should document intraspecific variation as comprehensively as possible, across sexes and different ontogenetic stages, with particular emphasis on diagnostic characters. Whenever feasible, morphological characters should be reported as ranges rather than as single values in order to capture variation amongst specimens. When extensive material is unvailable, detailed descriptions become crucial. Mouthparts are indispensable in both descriptions and re-descriptions and both the left and right mandibles should be illustrated and described.
3. Careful evaluation of synonymies and re-descriptions. Literature comparisons indicate that synonymies, historical species descriptions, subsequent re-descriptions and identifications, based on material from localities other than the type locality, should be treated with caution. Topotypic material is important in all such cases.

## Supplementary Material

XML Treatment for Stenothoe gallensis

97F5E629-3105-55AE-8B19-F968C5CAFD82 10.3897/BDJ.14.e199461.suppl1 Supplementary material 1 Figs S1, S2, S3 Data type images Brief description Supplementary material - Figs S1, S2, S3 *Stenothoe gallensis*. File: oo_1702148.docx https://binary.pensoft.net/file/1702148 Maria Lampa, Nafsika Papageorgiou, Dimitra Chatzivasileiou, Panagiotis D. Dimitriou, Stefania M. Manolaki, Ioannis Karakassis, Chatzigeorgiou Giorgos, Wanda Plaiti

7C31A0F6-6CBC-5AD5-BCF3-4065DDFE34D0 10.3897/BDJ.14.e199461.suppl2 Supplementary material 2 Table S1 Data type Excel spreadsheet with comparative table of diagnostic characters (morphological) Brief description Comparative table of diagnostic characters for the species currently recognised within the *Stenothoe gallensis* complex (including *Stenothoe lowryi* and, provisionally, *Stenothoe ogumi*) In quotation marks is the description as written by the author, spines = robust setae. Characters and descriptions highlighted in bold indicate features requiring particular attention, as potentially important diagnostic differences. File: oo_1698785.xlsx https://binary.pensoft.net/file/1698785 Maria Lampa, Nafsika Papageorgiou, Dimitra Chatzivasileiou, Panagiotis D. Dimitriou, Stefania M. Manolaki, Ioannis Karakassis, Chatzigeorgiou Giorgos, Wanda Plaiti

4773E4BB-6E84-52FB-90C3-9F8BD6493025 10.3897/BDJ.14.e199461.suppl3 Supplementary material 3 Supplementary material - Table S2 Data type morphometric variables Brief description Morphometric dataset of *Stenothoe gallensis* Walker, 1904 specimens. File: oo_1705148.xlsx https://binary.pensoft.net/file/1705148 Maria Lampa, Nafsika Papageorgiou, Dimitra Chatzivasileiou, Panagiotis D. Dimitriou, Stefania M. Manolaki, Ioannis Karakassis, Chatzigeorgiou Giorgos, Wanda Plaiti

5385F965-0389-5085-A5BF-3A8AA4E5C9D4 10.3897/BDJ.14.e199461.suppl4 Supplementary material 4 Table S3, S4 Data type literature and GBIF records Brief description Supplementary material Table S4: Mediterranean records of *Stenothoe gallensis* Walker, 1904 compiled from literature and GBIF.org. Supplementary material Table S5: Worldwide records of *Stenothoe gallensis* Walker, 1904 from 2015-2026, following the revision by Krapp-Schickel (2015), compiled from literature and GBIF.org. File: oo_1702149.xlsx https://binary.pensoft.net/file/1702149 Maria Lampa, Nafsika Papageorgiou, Dimitra Chatzivasileiou, Panagiotis D. Dimitriou, Stefania M. Manolaki, Ioannis Karakassis, Chatzigeorgiou Giorgos, Wanda Plaiti

## Figures and Tables

**Table 1.** Spearman rank correlations between morphometric variable pairs for male and female individuals of the *Stenothoe* specimens. Abbreviations are defined as follows: total length (TL); curvilinear dorsal body length (BL); curvilinear length of antennae (AL); body width (BW); gnathopod 2 propodus length (Gn2L); gnathopod 2 propodus width (Gn2W); gnathopod 2 dactylus length (dGn2L); length of setae on the of palmar margin of gnathopod 2 (sGn2L); and length of setae on inner margin of gnathopod 2 dactylus (dsGn2L).

	**Spearman's rank correlation coefficient ( rho)**	
**Morphometric variables compared**	**male**	**female**
TL vs. Telson robust setae	0.55*	0.69*
TL vs. U3 unpaired marginal robust setae	0.61*	0.60*
BL vs. AL	0.80*	0.67*
BL vs. BW	0.83*	0.79*
TL vs. Gn2L	0.76*	0.40*
TL vs. dGn2L	0.71*	0.63*
Gn2L vs. dGn2L	0.96*	0.81*
TL vs. Gn2W	0.68*	0.62*
Gn2L vs. Gn2W	0.93*	0.85*
sGn2L vs. Gn2L	0.78*	0.37*
TL vs. sGn2L	0.70*	0.26
*p-value < 0.05		

**Table 2.** Results of the Mann-Whitney U test for the different morphometric variables between different maturity stages (hyperadults and younger individuals). Abbreviations are defined as follows: total length (TL); curvilinear dorsal body length (BL); curvilinear length of antennae (AL); body width (BW); gnathopod 2 propodus length (Gn2L); gnathopod 2 propodus width (Gn2W); gnathopod 2 dactylus length (dGn2L); length of setae on the of palmar margin of gnathopod 2 (sGn2L); and length of setae on inner margin of gnathopod 2 dactylus (dsGn2L).

**Morphometric variable**	**U**	**p-value**
TL	576	9.989 × 10⁻⁸ *
BL	554	7.94 × 10⁻⁸*
AL	578	1.045 × 10⁻⁹*
BW	525.5	1.462 × 10⁻⁵*
Gn2L	579	7.198 × 10⁻⁸*
Gn2W	566	2.884 × 10⁻⁷*
dGn2L	584	4.151 × 10⁻⁸*
sGn2L	512.5	4.511 × 10⁻⁵*
Telson robust setae	436	0.0039*
U3 peduncle unpaired marginal robust setae	503.5	1.594 × 10⁻⁵*
*p-value < 0.05		
