# Supplementary material for: Current status of Stenothoe gallensis (Amphipoda, Stenothoidae): Towards the re-appraisal of the species complex with new records from Greece and challenges arising from intraspecific morphological variation
Source: Biodivers Data J. 2026 Jul 7;14:e199461. doi: 10.3897/BDJ.14.e199461 (PMC13370154; doi:10.3897/BDJ.14.e199461)
Supplement: Supplementary material 1 — Figs S1, S2, S3 [file bdj-14-e199461-s001.docx]

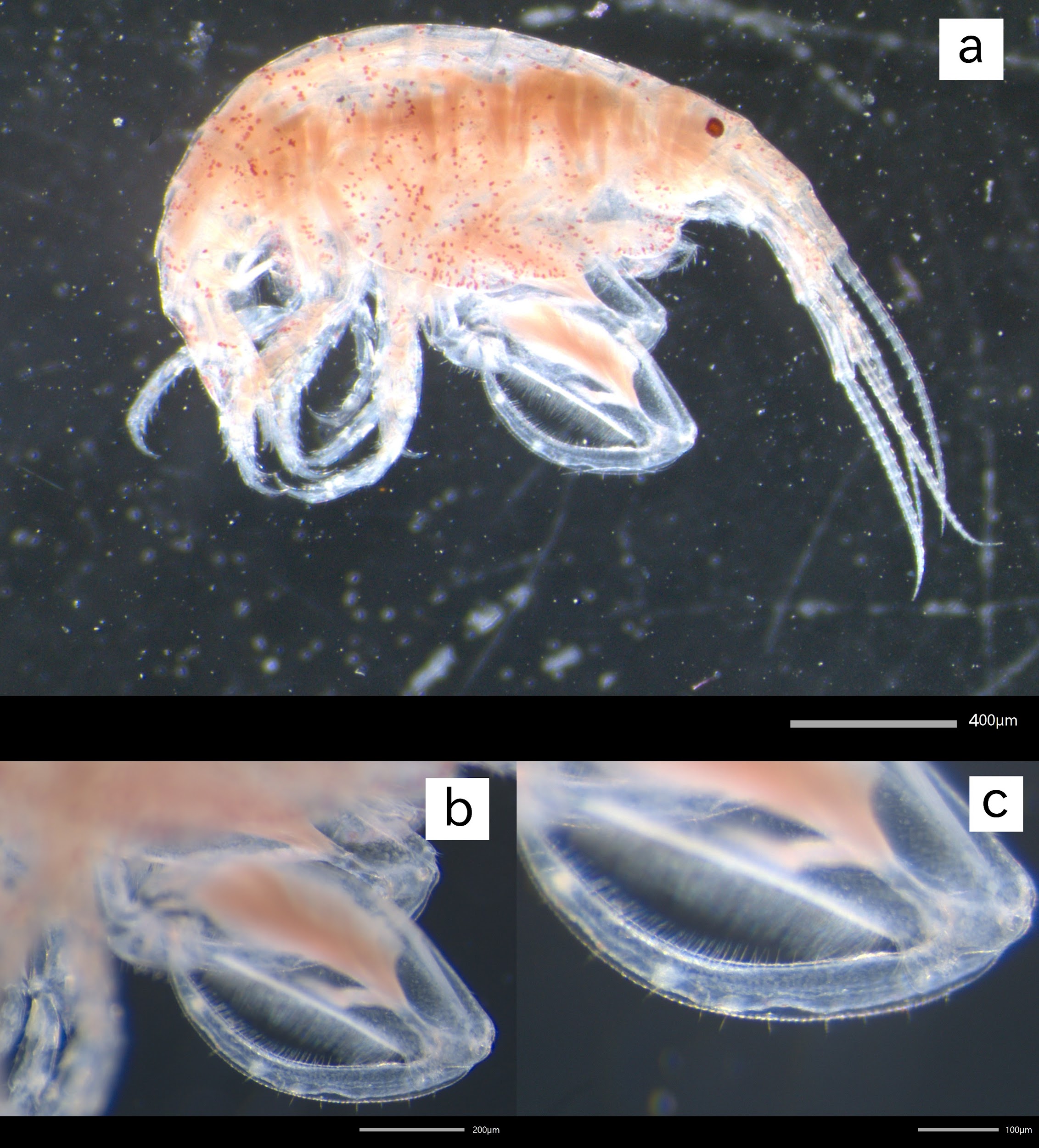


**Figure S1.** *Stenothoe gallensis*, (a) hyperadult male, (b) gnathopod 2, (c) detail of gnathopod 2 palmar margin, dactylus reaching proximal end of palmar margin.


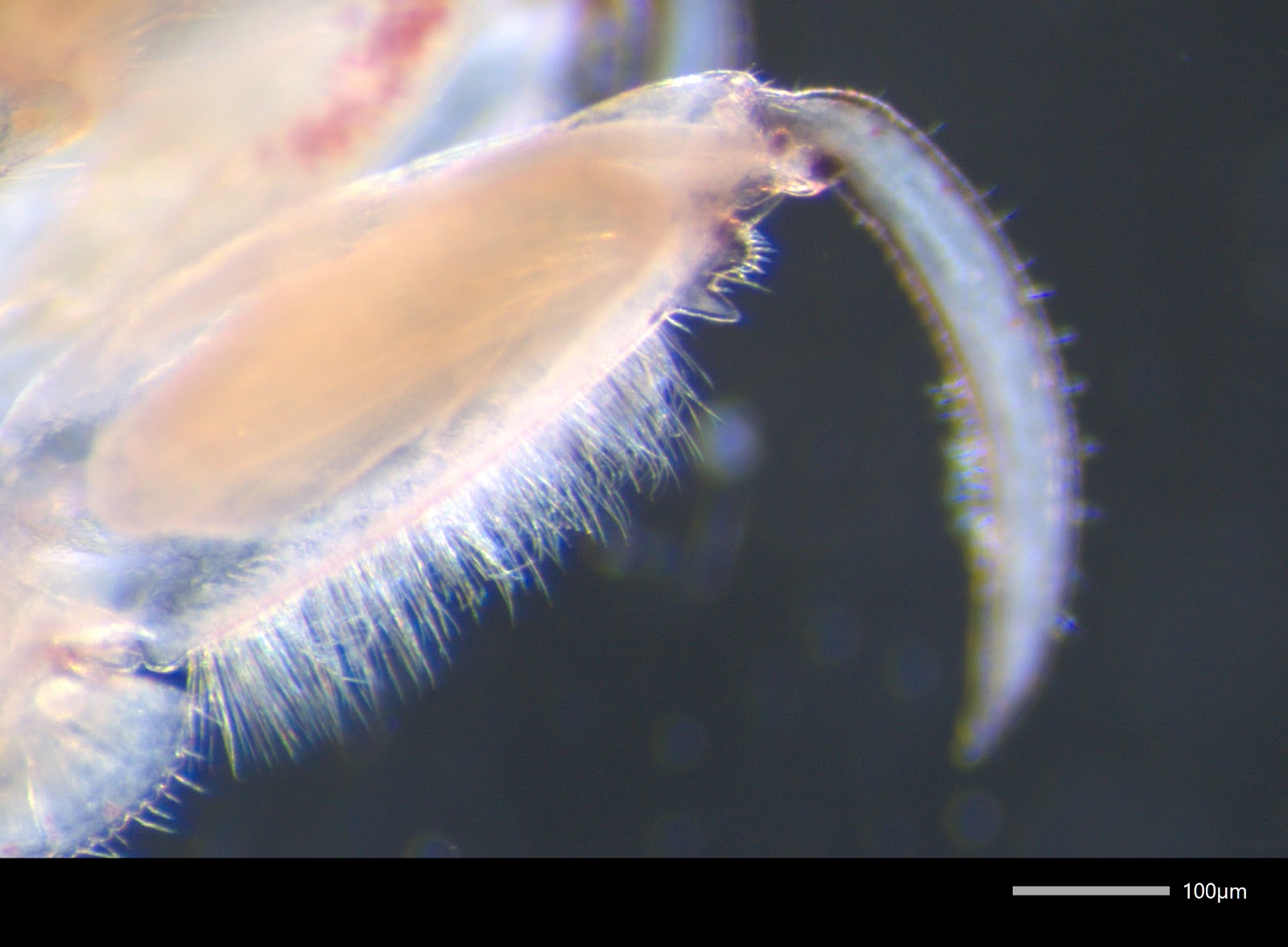


**Figure S2.** *Stenothoe gallensis*, hyperadult male gnathopod 2 palmar margin, specimen on the figure with dactylus not reaching proximal end of palmar margin.


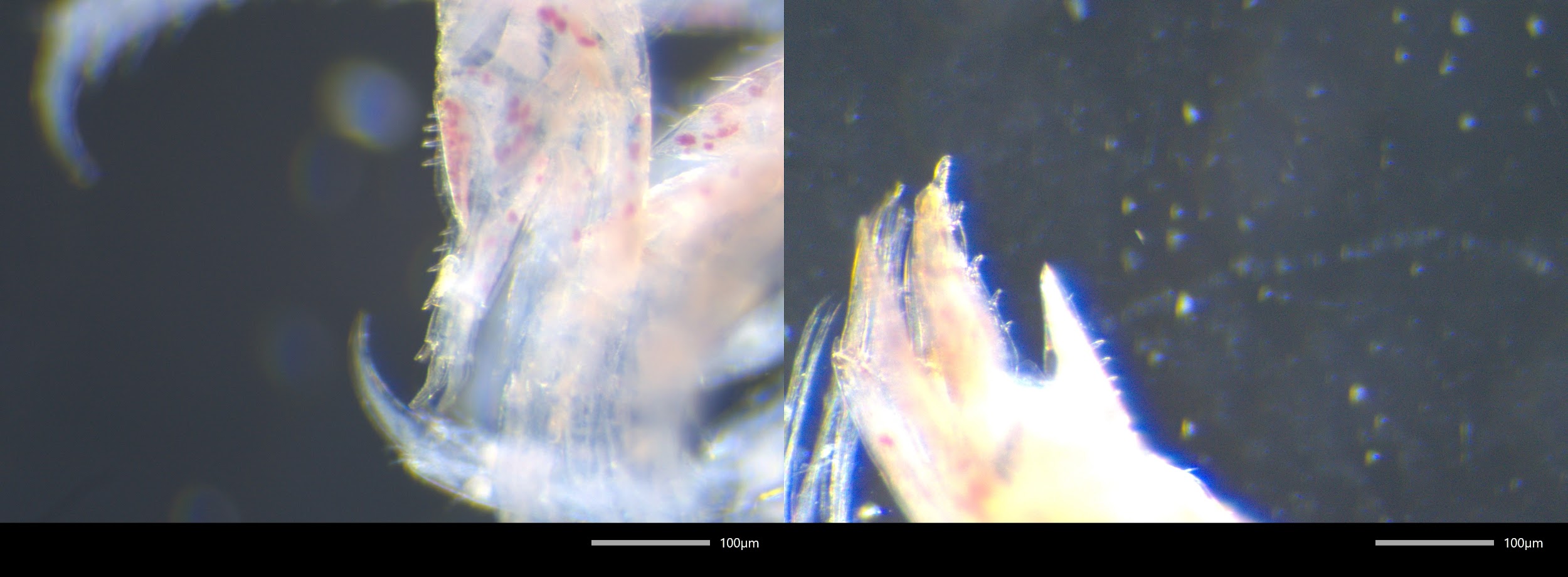


**Figure S3.** *Stenothoe gallensis*, detail of hyperadult male uropod 3. Specimen on the left with uropod 3 peduncle with 3 marginal robust setae and a pair of distal robust setae, telson with 4 robust setae on one side of the proximal margin. Specimen on the right with uropod 3 peduncle with 2 marginal robust setae and a pair of distal robust setae, telson with 4 robust setae on one side of the proximal margin increasing in size towards the distal end.
